# Dual role of exosomal circCMTM3 derived from GSCs in impeding degradation and promoting phosphorylation of STAT5A to facilitate vasculogenic mimicry formation in glioblastoma

**DOI:** 10.7150/thno.97057

**Published:** 2024-09-03

**Authors:** Chengbin Wang, Yingliang Liu, Zhenxing Zuo, Daming Cui, Yuzhen Xu, Li Li, Yang Jiang

**Affiliations:** 1 Department of Neurosurgery, Ruijin Hospital, Shanghai Jiaotong University School of Medicine, Shanghai 200025, China.; 2 Department of Neurosurgery, Shanghai Tenth People's Hospital, Tongji University School of Medicine, Shanghai 200072, China.; 3 Department of Rehabilitation, The Second Affiliated Hospital of Shandong First Medical University, Taian, 271000, China.; 4 Hospital for Chronic Neurological Diseases, Xi'an International Meidical Center Hospital Affiliated to Northwest University, Xi'an 710000, Shaanxi, China.

**Keywords:** glioblastoma stem cells, exosome, circRNA, vasculogenic mimicry, CNOT4, STAT5A

## Abstract

**Background:** Glioblastoma (GBM) is characterized by abundant neovascularization as an essential hallmark. Vasculogenic mimicry (VM) is a predominant pattern of GBM neovascularization. However, the biological functions of circRNAs prompting VM formation in GBM remains unclarified.

**Methods:** The circular RNA circCMTM3 was identified through high-throughput sequencing and bioinformatics analysis. The expression of circCMTM3 in exosomes in glioma tissues and cells was verified via RT-qPCR and FISH. In vitro and in vivo assays, such as EdU, MTS, Transwell, and tube formation assays were performed to investigate functional roles of circCMTM3. Meanwhile, in situ tumorigenesis assay were implemented to explore the influences of circCMTM3 on the GBM progression. Additionally, RNA pull-down, RIP, ChIP, and dual-luciferase reporter gene assays were executed to confirm the underlying regulation mechanism of circCMTM3.

**Results:** CircCMTM3, as a novel circular RNA, was packaged into exosomes derived from glioblastoma stem cells (GSCs), which facilitates the phenotypic transition of differentiated glioma cells (DGCs) to VM. Mechanistically, exosomal circCMTM3 is internalized by DGCs and disrupt the ubiquitination degradation of STAT5A and STAT5B by E3 ubiquitin ligase CNOT4. Additionally, through molecular scaffold function of circCMTM3, STAT5A is activated and triggers transcriptional regulation of target genes including the pro-vasculogenic factor CHI3L2 and the RNA-binding protein SRSF1. Subsequently, circCMTM3/STAT5A/SRSF1 positive feedback loop sustainably enhances VM formation and accelerates tumor progression in GBM.

**Conclusion:** Exosomal circCMTM3 possessing growth factor-mimetic property activates the JAK2/STAT5A pathway via non-canonical manner, and promotes VM formation in GBM. The molecular communications between GSCs and DGCs offers a therapeutic strategy for targeting the neovascularization of GBM.

## Background

Glioblastoma (GBM), a rare and high-grade malignancy of central nervous system with an extremely high fatality rate in both adults and pediatrics, poses enormous challenges in terms of surgical treatment and adjuvant radiochemotherapy^1^. Despite advancements in biotechnology, our understanding of the pathogenic mechanisms and treatment resistance of GBM has been reassessed, it is still an urgent need to improve clinical outcomes for patients.

Plasticity of cellular phenotypes has been identified as a major contributor to intra- and inter-tumoral heterogeneity as well as treatment resistance in GBM. Increasing evidence indicates that glioblastoma cells exhibit remarkable intrinsic plasticity to acclimate to dynamic microenvironment^2^. Similar to neural stem cells differentiating into neurons and glial cells, GBM stem cells (GSCs) could generate a variety of differentiated morphologies with astrocytic or neuronal phenotypes^3^. Several molecules regulating the switch between GSCs and non-GSCs have been reported, involving nitric oxide promoting the activation of the Notch signaling pathway resulting in the emergence of GSCs phenotypes^4^, and bone morphogenetic protein 4 (BMP4) inducing astroglial-like differentiation and quiescence^5^. Limiting the adaptive capacity of GBM, including targeting plasticity regulators and stemness-differentiation phenotype switching mechanisms, is a worthwhile investigation, which could be therapeutically exploited to overcome treatment resistance.

Vasculogenic mimicry (VM) is a form of angiogenesis independent of vascular endothelial cells and observed in many malignant solid tumors. Therefore, VM is considered as an emerging model of neovascularization in invasive tumors, supplying blood and nutrient to tumor proliferation. In a period of VM formation, cancer cells with endothelial-like characteristics arrange themselves into tubular structures, which deliver nutrients and oxygen-rich erythrocytes into tumor tissues^6^. The tubular structures composed of CD31/CD34-negative and PAS-positive cells, along with the presence of erythrocytes within, are commonly used as identifying criteria for VM. VM is typically observed in highly invasive, metastatic, and advanced malignant tumors, indicating poor prognosis for patients^7^.

Exosomes, the smallest components of extracellular vesicle (EVs), play a crucial role in the tumor microenvironment (TME) by regulating cytoarchitecture including tumor cells, immunocytes, stromal cells, endotheliocyte, and others. They carry bioactive molecules such as proteins, nucleic acids, lipids, and metabolites, which have extensive effects on the immune system, tumor metabolism, and drug resistance, thereby influencing the malignant process of tumors^8^. There is growing evidence that exosomes play a prominent role in facilitating cell-to-cell communication between parent cells and recipient cells^9^. CircRNAs, a type of non-coding RNA, serves a critical regulator function in tumorigenesis and progression^10^. These circRNAs have unique advantages, such as abundant content and relative stability benefiting from the circular structure, as well as the availability to be packaged into exosomes for intercellular transmission. It leads to the formation of "exosomal circRNAs," which are emerging as pivotal biomarkers and therapeutic targets for cancer^11^. Recent research shows that the expression of various exosomal circRNAs is often dysregulated in the TME. In glioma, circNEIL3 is encapsulated into exosomes by hnRNPA2B1 and transmitted to infiltrated tumor-associated macrophages (TAMs), thereby inducing immune suppression^12^. In addition, exosomal circKIF18A derived from GBM-associated microglia promoted angiogenesis in GBM by regulating FOXC2 nuclear translocation in human brain microvascular endothelial cells (hBMECs)^13^.

In this research, through high-throughput sequencing, a noteworthily upregulated circRNA, hsa_circ_0008450 (termed circCMTM3), was verified enriched in GSCs-derived exosomes (GDEs). Overexpression of circCMTM3 correlates with glioma grade and poor prognosis in a clinical cohort containing 70 glioma cases. We demonstrated that circCMTM3 was observably up-regulated in GDEs. Exosomal circCMTM3 was proved to be delivered to differentiated glioma cells (DGCs), further promoting invasion, migration, and VM in DGCs to accelerate the malignant progression of GBM, through in vivo and vitro assays. Our study aimed to indicate that exosomal circCMTM3 promotes VM in GBM and holds promise as a novel target for anti-vasculogenic therapy.

## Methods

### Glioma tissue specimen collection and ethical approval

Seventy glioma tissue specimens were collected from neurosurgical resections of glioma patients in the Department of Neurosurgery at the Shanghai Tenth People's Hospital during 2015-2019. Each specimen was histopathologically assessed and then classified in accordance with the 5th edition of the World Health Organization (WHO) classification of central nervous system tumors. Additional ten specimens from normal brain tissue were obtained as comparison. All patients enrolled provided the written informed consent. This study complied with all relevant ethical guidelines and was approved by the Ethics Committee of the Shanghai Tenth People's Hospital.

### Cell culture and reagents

Detailed clinical information of the patients-derived GSC01 and GSC03 cell lines involved in this study is recorded in Table S1. All patient-related research was conducted in accordance with the Helsinki Declaration. The isolation, culture, and identification protocols of GSCs from GBM tissue were described in previous studies^14^. In summary, immunofluorescence staining was performed on neurospheres formed by GSCs to detect the expression of stemness markers CD133 and NESTIN (Figure S1A). The morphology of DGCs generated under serum-containing culture conditions was observed and recorded using a light microscope (Figure S1B). Additionally, the expression of GFAP and β III-tubulin in DGCs was assessed through immunofluorescence staining (Figure S1C). Finally, Western blot analysis was conducted to examine the expression of stemness markers in both GSCs and the corresponding DGCs (Figure S1D). All GSCs cell lines derived from patients were subjected to short tandem repeat (STR) DNA analysis and mycoplasma detection. During the experimental procedure, DGCs were treated with Stafia-1 (20 μM), a specific inhibitor of STAT5A, purchased from Selleckchem (Houston, Texas, USA).

### Exosome extraction, purification, and storage

To isolate and purify exosomes derived from GSCs and DGCs, we collected the culture supernatant of GSCs cultured for 48 h and the exosome-free culture supernatant of DGCs cultured for 48 h. Subsequently, the collected supernatants were sequentially centrifuged at 2000 g for 10 mins, 10000 g for 30 mins, and 120000 g for 70 mins at 4 °C. This allowed us to obtain purified exosomes, which were resuspended in PBS and stored at -80 °C for further analysis. An experimental protocol for obtaining exosomes from glioma and adjacent normal brain tissue sources was described in a recent study^15^. Briefly, the tissue samples were washed with PBS on ice, minced, and homogenized. The Tumor Dissociation Kit (Miltenyi biotec, Germany) was used to digest and separate the tissue into a single-cell suspension. Under 4 °C conditions, the suspension was centrifuged at 500 g for 10 mins and 2000 g for 10 mins to obtain the cell-free supernatant. The supernatant was then subjected to stepwise centrifugation, as described before, following the protocol for purifying extracellular vesicles, to obtain purified tissue-derived exosomes.

### CircRNA sequencing

Total exosomal RNAs from GSCs and DGCs were isolated using the Total Exosome RNA and Protein Isolation Kit (Invitrogen, Carlsbad, CA, USA). After extraction, ribosomal RNAs (rRNAs) were depleted to retain messenger RNAs (mRNAs) and non-coding RNAs (ncRNAs). The enriched mRNAs and ncRNAs were fragmented and reverse transcribed into cDNA using random primers. Subsequently, the cDNA fragments were purified using a QiaQuick PCR extraction kit (Qiagen, Venlo, The Netherlands), end-repaired, poly(A) conjugated, and ligated to Illumina sequencing adaptors. After degradation of second-strand cDNA and PCR amplification, the products were sequenced using an Illumina NovaSeqTM 6000 platform. For circRNA quantification, back-spliced junction reads were normalized to reads per million mapped reads (RPM) and analyzed with find_circ for circRNA identification. Differentially expressed circRNAs across samples or groups were identified using the edgeR package, with selection criteria of fold change >1 and a P value < 0.05.

### Transmission electron microscopy analysis

The isolated and purified exosomes were transported to the School of Life Sciences and Technology, Tongji University, for transmission electron microscopy (TEM) analysis and imaging using a Hitachi microscope from Japan. Prior to analysis, the exosomes were washed with PBS and fixed with 2.5 % glutaraldehyde.

### Nanoparticle tracking analysis

After resuspension of the exosomes at a concentration of 2×10^9^ particles/ml, the concentration and size of the exosomes were evaluated using a Nanosight LM10 instrument (Malvern, Framingham) under 25 °C and with 488 nm laser conditions. The NTA v3.1 software (Malvern, Framingham) was used for result analysis and data recording.

### Exosome tracking assays

The PKH26 Red Fluorescent Cell Linker Midi Kit (Sigma-Aldrich, Germany) was used for labeling exosomes with PKH26 dye. Subsequently, PKH26-labeled exosomes were used to intervene target cells at a concentration of 10 μg/mL for 6 h at 37 °C with a 5 % CO_2_ atmosphere. After washing, fixation, and DAPI staining, visualization and image acquisition were performed using an LSM900 confocal microscope (ZEISS, Germany).

### RNA fluorescence in situ hybridization (FISH)

The FISH assay was conducted using the FISH Tag™ RNA Multicolor Kit (Invitrogen, USA) according to the manufacturer's instructions. Cy3-labeled oligonucleotide probes complementary to the junction sequence of circCMTM3 were synthesized by Gene-Chem (Shanghai, China). Fluorescent images were acquired using a precision imaging system consisting of an ECLIPSE Ts2 fluorescence microscope, Digital Sight 10 camera, and NIS-Elements software (Nikon, Japan).

### Construction and transfection of lentiviral vectors and plasmids

The lentiviral vectors used for gene overexpression and silencing in this study were prepared by Gene-Chem (Shanghai, China). Stable cell lines were selected using puromycin (Gibco™, Thermo Fisher Scientific, USA). The RNAi sequences utilized in this study are listed in Table S2. The plasmids used for transient expression were also provided by Gene-Chem and transfected into cells with the aid of lipofectamine 3000 (Invitrogen, USA) following the instructions provided with the reagent kit. Transfection efficiency was confirmed by qPCR and western blotting assays.

### Quantitative real‑time PCR (RT‑qPCR) assay

Total RNA was isolated from cells, tissues, and exosomes utilizing TRIzol (Invitrogen, USA) as per the manufacturer's guidelines. The construction of the cDNA library was carried out utilizing the Prime-Script RT Master Mix Kit (TaKaRa, Kyoto, Japan). RT-qPCR was conducted on the Mx-3000P Quantitative PCR System (Applied Biosystems, USA) utilizing the SYBR Green Master Mix Kit (TaKaRa). The primers utilized in this study were obtained from Sangon Biotech (Shanghai, China), and their sequences are provided in Table S3.

### RNase R assay

As mentioned previously, a total of 10 μg RNA extracted from GSCs was subjected to incubation with 40 U of RNase R (Epicentre Technologies, Madison, WI, USA) at 37 °C for a duration of 30 mins. Subsequent to completion of the reaction, RT-qPCR were used to evaluate the expression levels of both linear RNAs and circular RNAs.

### Western blotting assay

Each group of DGCs were lysed in RIPA buffer supplemented with protease and phosphatase inhibitors (Beyotime Biotechnology, Beijing, China) on ice, and centrifuged at 13,000 g for 15 mins at 4 °C. The proteins were quantified, denatured, subjected to SDS-PAGE, and subsequently transferred onto PVDF membranes. After blocking in 5 % non-fat milk, membranes were incubated with the primary antibody overnight at 4 °C, and then were immunoblotted with the HRP-conjugated secondary antibodies at room temperature for 1 h. The bands were visualized by the chemiluminescence ECL kit (YEASEN, Shanghai, China) and a chemiluminescence imaging system (Tanon, Shanghai, China), with intensity quantified by ImageJ (National Institutes of Health, Bethesda, MD, USA). Detailed information regarding the antibodies employed in this study can be found in Table S4.

### Protein stability assessment

DGCs were treated with MG132 (50 μM) (Sigma-Aldrich) for 6 h. Following protein extraction, immunoblotting was conducted to determine the presence of the target proteins. Furthermore, DGCs were intervened with 50 μg/ml cycloheximide (Sigma-Aldrich), and subsequently lysed at 0 h, 2 h, 4 h, 8 h, and 12 h to collect proteins for protein half-life assessment.

### RNA pull-down assay

The RNA pull-down assay was conducted via employing the Pierce™ Magnetic RNA-protein pull-down kit (Thermo Fisher Scientific, USA) following the instructions. In brief, the lysate obtained from DGCs was incubated with biotinylated circCMTM3 and anti-sense probes. Subsequently, streptavidin-conjugated magnetic beads were added to the system. After elution and purification, the enriched proteins were separated via SDS-PAGE gel electrophoresis and visualized using a silver staining kit (Beyotime Biotechnology) for mass spectrometry analysis. The expression levels of the interacting proteins were further verified using western blotting. The silver-stained protein band was excised for digestion and subsequently analyzed using a QExactive mass spectrometer (Thermo Fisher Scientific).

### Co‑immunoprecipitation

The co-immunoprecipitation (Co-IP) assays were performed utilizing the Pierce Classic Magnetic IP/Co-IP Kit (Thermo Fisher Scientific) to investigate protein interactions in DGCs. Briefly, DGCs were lysed using IP lysis buffer supplemented with protease and phosphatase inhibitors. Following the manufacturer's protocol for the Co-IP kit, lysates of DGCs were incubated with antibodies conjugated to magnetic beads at 4 °C overnight. Subsequently, immunoprecipitates and whole-cell lysates were collected for western blot analysis assessing the proteins interaction capabilities.

### Ubiquitination assay in vivo

The ubiquitination assay was conducted following established protocols^14^. In brief, DGCs from various experimental groups were transfected with Flag-STAT5A, Flag-STAT5B, HA-Ub-WT, HA-Ub-K6R, K11R, K48R, or K63R plasmids (obtained from Gene-Chem, Shanghai, China) using Lipofectamine 3000 (Invitrogen) prior to treatment with MG132 for 6 h. DGCs lysates were then prepared using an IP buffer and utilized for the ubiquitination assay. Immunoprecipitation of proteins was performed using an antibody against the Flag tag (Abcam Technology, Cambridge, UK), followed by the detection of ubiquitination through immunoblotting with anti-HA or anti-Ubiquitin antibodies (Abcam).

### RNA immunoprecipitation (RIP) assay

The RIP assay was conducted using the EZ-Magna RIP kit (Millipore, Germany) following the manufacturer's instructions. In brief, DGCs were lysed with PIP buffer and then incubated with magnetic beads conjugated with anti-CNOT4, anti-STAT5A, anti-STAT5B, anti-JAK2 and anti-SRSF1 antibodies. The complexes of proteins and RNAs were immunoprecipitated, and RNAs were obtained after treatment with proteinase K, followed by washing and purification steps. The expression level of circCMTM3 was assessed using RT-qPCR. An IgG antibody from Abcam was utilized as a negative control in this experimental assay.

### Dual luciferase reporter gene assay

The objective of the dual-luciferase reporter gene assay was to investigate the transcriptional regulation of STAT5A on CHI3L2 and SRSF1. The full nucleotide sequences of the CHI3L2 and SRSF1 promoters, both wildtype (CHI3L2-WT, SRSF1-WT), and mutant clones of the promoter regions with STAT5A binding site mutations (CHI3L2-MT, SRSF1-MT) were inserted into the pGL3 vectors. DGCs from different groups with treatment by GDEs or Stafia-1were plated in a 24-well plate and co-transfected with pRL TK (renilla luciferase reporter vector) and the pGL3 vector plasmids (or CHI3L2-WT, CHI3L2-MT, SRSF1-WT, SRSF1-MT plasmids). After 24 h, the luciferase and renilla luciferase activities were measured and analyzed using a dual-luciferase reporter gene assay kit (Beyotime) following the manufacturer's instructions.

### Chromatin immunoprecipitation (ChIP) assays

Chromatin immunoprecipitation (ChIP) assays were conducted utilizing the ChIP Assay Kit (Beyotime) following the protocol provided by the manufacturer. Specifically, anti-STAT5A antibody was employed for immunoprecipitation of chromatin complexes, with Histone H3 antibody from Cell Signaling Technology used as a positive control and IgG antibody from Abcam utilized as a negative control. Subsequently, DNA was extracted and purified from the immunoprecipitated complexes, and RT-qPCR was performed for enrichment analysis. The primers used for ChIP qPCR can be found in Table S5.

### RNA dynamic assay

DGCs with knockdown or overexpression of SRSF1 were treated with actinomycin D (Sigma-Aldrich) at a final concentration of 5 μg/mL, and incubated with different types of DGEs simultaneously. Total RNA from DGCs was extracted at 6, 12, 18, 24, 30, and 36 h. The content of circCMTM3 was detected by qPCR to calculate the degenerated rates and half-life (t_1/2_).

### Cell proliferation assay

Cell proliferation was assessed through MTS and EdU assays. DGCs were plated in 96-well plates at a density of 10^3^ cells per well, and 20 μl of MTS (Promega, Madison, WI, USA) was added to each well at 24, 48, 72, 96, and 120 h. Following a 3-h incubation period, the absorbance at 495 nm was measured with a UV spectrophotometer (Thermo Fisher Scientific). Additionally, the EdU assay was conducted using the EdU assay kit (Beyotime) following standard protocols. Briefly, DGCs were seeded at a density of 10^4^ cells per well in 24-well plates and cultured for 24 h. Subsequently, 10 μM of EdU reagent was added to each well and incubated for 2 h. After steps involving fixing, EdU incorporation, and nuclear staining of the DGCs, the experimental results were imaged using an ECLIPSE Ts2 fluorescence microscope (Nikon, Japan). Finally, the percentage of EdU-positive cells was quantified.

### Cell migration assay

Migration of DGCs was assessed by the HoloMonitor M4 culture system (Phase Holographic Imaging PHI AB, Lund, Sweden). Briefly, cells were seeded into a six-well plate at a density of 10^4^ cells/ml. After cultured and incubated with exosomes, cells were photographed by HoloMonitor M4 culture system every 2 h for a total of 12 h. Cell image of each group was recorded and stored at the last point in time, and then the cellular movement trajectory was simulated and analyzed.

### Tube formation assay

The tube formation assay was performed as previously described. Briefly, 24-well culture plate was coated with 100 μl Matrigel (BD Biosciences) per well and then incubated at 37 °C for 30 mins. DGCs from different groups were inoculated in the plates at 2 × 10^4^ cells/well for 24 h and formed tubular structures were imaged by phase-contrast microscopy (Nikon), and Image J software was used to calculate the total number of branches and tubule lengths.

### Transwell assay

For the transwell invasion assay, 2 × 10^4^ DGCs were added into the superior chamber (Corning, Corning, NY, USA) after the addition of 100μl Matrigel (BD Biosciences) in it. The medium added with 20 % fetal bovine serum was injected to the lower chamber. After incubation for 24 h, cells possessed invasive properties were fixed stained with crystal violet (Beyotime, Biotechnology), then imaged by Nikon microscope for counting.

### IF and IHC assays

Immunofluorescence (IF) staining of DGEs were conducted in the confocal dish. Briefly, cells were fixed with 4 % PFA for 15 mins and blocked for 1 h. Petri dishes were incubated with primary antibodies at 4 °C overnight and fluorescent secondary antibodies for 1h at 25 °C. After washing with PBS, antifade solution containing DAPI (Abcam) was used for mounting. Images were captured with a confocal microscope (ZEISS). For immunohistochemistry (IHC) of brain/tumor sections, IHC labeling kit (Immunoway Biotechnology, USA) was utilized follow the manufacturer's instruction. For the double-staining of VM structures, tumor sections were stained by IHC for CD31 and then incubated in periodic acid and Schiff's fuchsin-sulfite reagent for PAS staining after antigen retrieval. PAS^+^-CD31^+^ tubular structures were defined as endothelial vessels while PAS^+^-CD31^-^ were VM vessels. VM lumens were enclosed by glioma cells rather than endothelial cells. The VM score was calculated as the ratio of VM vessels/total vessels and expressed in percent^16^. Images of sections are acquired through upright microscopy (Leica, DM4000B). The antibodies used for IF/IHC is provide in Table S4.

### In situ tumorigenesis assay

The animal experimental protocol involved in this study was approved by the Shanghai Tenth People's Hospital Experimental Animal Ethics Committee. Six-week-old male BALB/C nude mice were purchased from the Model Animal Research Center of Nanjing University (Nanjing, China). All mice were housed under specific pathogen-free conditions at the Shanghai Tenth People's Hospital Animal Center. As previously described^14^, each experimental group comprised 5 mice for establishing the glioblastoma xenograft model. Briefly, following anesthesia, patient derived GSCs were injected into the striatum (relative to bregma: -0.2 mm, lateral ±2 mm, depth 2.6 mm from the dura mater) of nude mice brains at a rate of 0.1μL per minute using a 10μL Hamilton syringe (at a density of 5×10^4^ cells in a 5 μL solution) using a stereotactic device. The needle was held stationary for over 5 minutes after injection. Six days later, glioma-bearing mice received intravenous injections of different types of GDEs (30 μg per mouse per injection) every 3 days for a consecutive 10 days. Stafia-1, Dynasore and bevacizumab began intravenous injections respectively starting from the 6th day post GSCs implantation and continued for 10 days. Distress or death signs of each group mice was observed and recorded daily. The overall survival times of mice were detected through Kaplan-Meier survival analysis. The mice brains were collected after perfusion with 4 % paraformaldehyde for H&E and immunohistochemistry (IHC) staining. Tumor volume was calculated using the formula: V = (D×d^2^)/2, where D is the maximum diameter, and d is the minimum diameter.

### Bioinformatics analysis

The biological information regarding CircCMTM3 was retrieved from circBase (http://www.circbase.org), Cancer-Specific CircRNA Database (CSCD, https://gb.whu.edu.cn/CSCD/), and circInteractome (http://circinteractome.nia.nih.gov). Transcriptomic data from the TCGA glioma cohort were accessed through the GDC Data Portal (https://portal.gdc.cancer.gov/). Similarly, RNA-seq data from the Chinese Glioma Genome Atlas (CGGA) were obtained from the CGGA database (http://www.cgga.org.cn/). Gene Set Enrichment Analysis (GSEA, http://www.broadinstitute.org/gsea/index.jsp) and Gene Set Variation Analysis (GSVA) were utilized to investigate the enrichment of angiogenesis processes or signaling pathways based on differential STAT5A expression levels.

### Molecular Docking Analysis

To investigate the relationship between CNOT4 and STAT5A as well as STAT5B, rigid protein-protein docking was performed using GRAMM-X (http://gramm.compbio.ku.edu/). The protein structural domains of CNOT4, STAT5A, and STAT5B were obtained from the AlphaFold Protein Structure Database (https://alphafold.ebi.ac.uk/). Protein-protein interactions were further analyzed and visualized using Pymol (Version 2.4) and PDBePISA (https://www.ebi.ac.uk/pdbe/pisa/). Molecular docking analysis and visualization of circCMTM3 and CNOT4 were conducted using HDOCK (http://hdock.phys.hust.edu.cn/). Additionally, the secondary and tertiary structures of circCMTM3 were predicted and obtained using cRNAsp12 17 and CircularSTAR3D 18 respectively.

### Statistical analyses

Statistical tests were performed using R version 4.2.1 and GraphPad Prism version 9.0. T-tests were employed for pairwise comparisons, and one-way ANOVA was used for multiple comparisons. For non-parametric data, statistical significance was determined by the Wilcoxon test. Correlation between groups was assessed by Pearson's correlation. Survival analysis was performed by the Kaplan-Meier curve and log-rank test. P-values < 0.05 were accepted as statistically significant, provided in Figure legends. (*p < 0.05; **p < 0.01; ***p < 0.001).

## Results

### Upregulated exosomal circCMTM3 driven from GSCs is associated with poor prognosis of glioma patients

**Figure 1:**
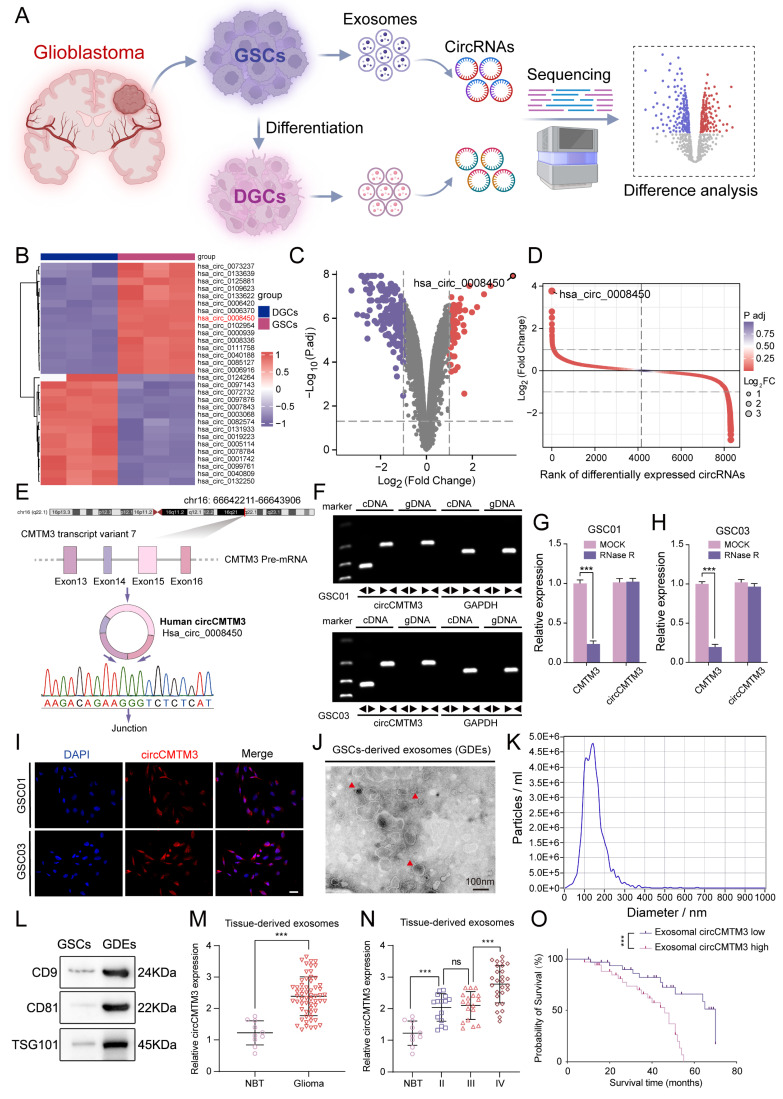
** Exosomal circCMTM3 is upregulated in GBM and correlated with poor prognosis. A** Schematic diagram of the acquisition and analysis of exosomal circRNAs data from patient-derived GSCs and DGCs. **B** Heatmap displaying the z-scores value of circRNAs differentially expressed in exosomes derived from GSCs and DGCs respectively. **C** Volcano plot depicting the log_2_ (fold change) of circRNAs in the two types of exosomes mentioned above. Grey dashed lines represent the cutoff value, which is P. adj value < 0.05 and | log_2_(fold change) |> 1. Downregulated (purple) and upregulated (red) circRNAs in exosomes are color-coded. **D** Rank of differentially expressed exosomal circRNAs according to values of P. adj and log_2_FC. **E** A schematic representation detailing the genomic characteristics of circCMTM3 (hsa_circ_0008450). The upper panel depicted the genomic location of the parental gene with its exons structure and the back-splicing site, as identified through Sanger sequencing, is displayed at the bottom of the panel. **F** Agarose gel electrophoresis of RT-qPCR assays showing the expression of circCMTM3 amplified from templates of GSC01 (upper panel) and GSC03 (lower panel) using divergent and convergent primers. **G, H** The RNA expression levels of circCMTM3 and CMTM3 in GSC01 (G) and GSC03 (H) after RNase R treatment. **I** Representative fluorescence images of in situ hybridization detection of circCMTM3 expressional location in GSC01 and GSC03. Scale bar = 50 μm. **J, K** The transmission electron microscopy analysis (J) and nanoparticle tracking analysis (K) displaying the morphologic characteristics and size distribution of GDEs. Scale bar = 100 nm. **L** Western blotting analysis of exosomal markers CD9, CD81 and TSG101 in GDEs. **M** The circCMTM3 expression level in glioma (n = 70) and normal brain (n = 10) tissues-derived exosomes. **N** The circCMTM3 expression difference in gliomas tissues-derived exosomes with different malignant grades. **O** Kaplan-Meier survival curve for all glioma patients with high and low exosomal circCMTM3 expression. Data are presented as means ± SD (three independent experiments). *p < 0.05; **p < 0.01; ***p < 0.001; ns, no significance.

To explore the significant role of exosomal circRNAs in the mechanism of GBM stemness-differentiated cell phenotypic transition, high-throughput sequencing was performed on exosomal circRNAs originated from three GSCs and their corresponding DGCs (Figure 1A. Differential analysis revealed that 92 circRNAs were upregulated in GDEs while 195 were downregulated compared to DGCs (Figure 1B-D). CircCMTM3 (circBase ID: Has_circ_0008450, chr16: 66642211-66643906) was selected as the candidate with the most distinct differential expression for subsequent analysis. CircCMTM3 is produced by reverse splicing of exons 13-16 of its precursor gene CMTM3 pre-mRNA transcript variant 7 and was confirmed by Sanger sequencing (Figure 1E). Amplification of circCMTM3 using divergent primers could only be achieved by reverse transcription of cDNA from GSC lines GSC01 and GSC03, but not from genomic DNA (gDNA) (Figure 1F). In addition, treatment with RNase R led to a remarkable decrease of linear mRNA of CMTM3, without affecting the content of circCMTM3 due to its closed-loop structure (Figure 1G-H). Furthermore, FISH experiments showed abundant expression of circCMTM3 in GSCs, of cytoplasmic distribution (Figure 1I), which coincided with its high expression in GDEs. It suggested that circCMTM3 might primarily mediate communication between GSCs and other cells in GBM microenvironment through extracellular secretion. Besides, detailed analysis of the characteristics of GDEs revealed cup-shaped morphology and a size distribution of 30-150nm, as observed by transmission electron microscopy and nanoparticle tracking analysis (NTA). Moreover, GDEs exhibited high expression of exosome markers, including CD9, CD81, and TSG101 (Figure 1J-I). Moreover, the expression level of circCMTM3 in exosomes derived from GBM tissue was notably higher than that in normal brain tissue (Figure 1M). Additionally, the expression level of exosomal circCMTM3 was positively correlated with glioma grades (Figure 1N). Kaplan-Meier survival analysis stratifying GBM patient cohorts based on the relative expression level of exosomal circCMTM3 compared to the median demonstrated that patients with high expression of exosomal circCMTM3 had shorter overall survival compared to those with low expression (Figure 1O). In conclusion, the above findings suggest that circCMTM3 is abundantly packaged in GDEs, and the overexpression of exosomal circCMTM3 is associated with poor prognosis in glioma patients.

### Exosome-packaged circCMTM3 can be internalized into DGCs and stably express

**Figure 2:**
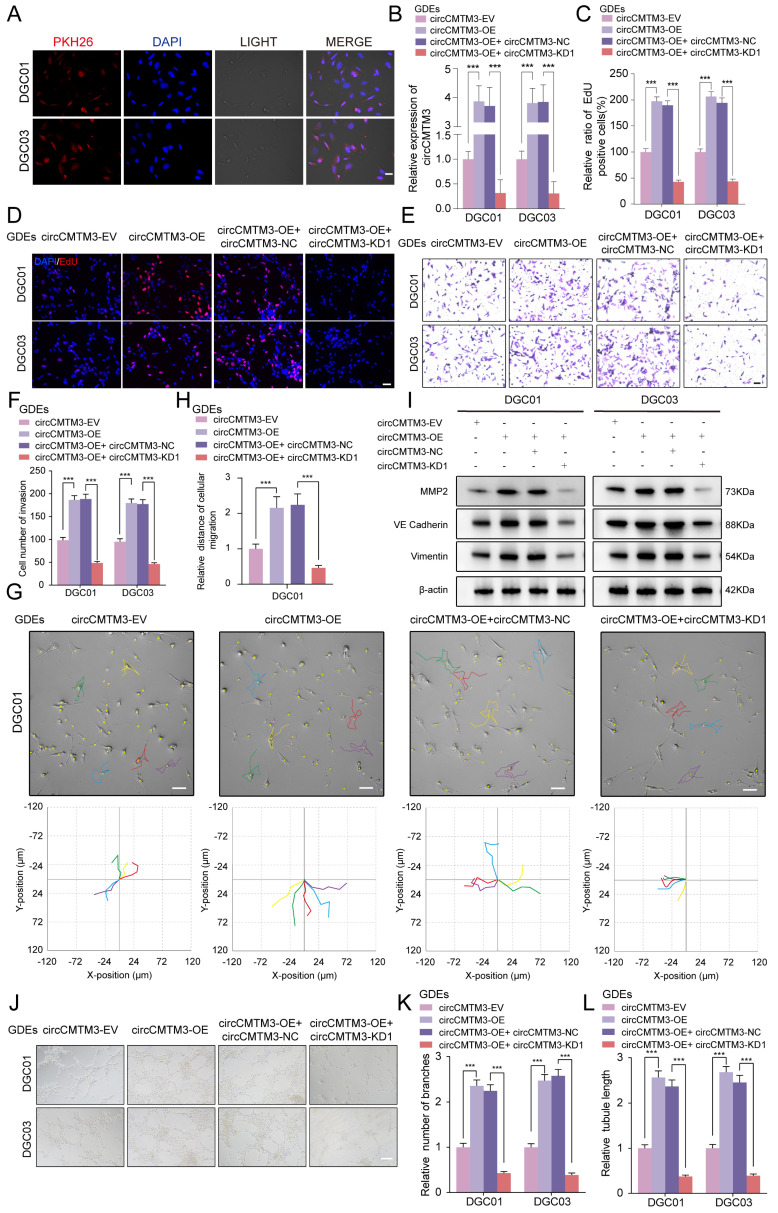
** Exosomal circCMTM3 can be internalized and expressed in DGCs to promote VM formation in vitro. A** Representative fluorescence images of DGCs after incubating with PKH26-labeled GDEs. Scale bars = 50 μm.** B** RT-qPCR analysis of circCMTM3 expression in DGCs treated with different groups of GDEs.** C** Quantification of the EdU positive cells of DGCs in different GDEs treatment groups.** D** Representative images of EdU assays showing the proliferation of DGC01 after incubating with different groups of GDEs. Scale bars = 100 μm.** E, F** Representative images (E) and quantification (F) of the Transwell assay of DGCs treated with different groups of GDEs. Scale bars = 50 μm.** G** The migration ability of DGC01 is detected by HoloMonitor and visualized in Hstudio (n = 5) in different GDEs treatment groups. Scale bars = 50 μm.** H** Quantification of relative migration distance of DGCs by monitor visualization. **I** Western blotting analysis of VM markers MMP2, VE-Cardherin and Vimentin.** J-L** Representative images (J) and quantification (K, L) of the tube formation assay of DGCs treated with different groups of GDEs. Scale bars = 100 μm. Data are presented as means ± SD (three independent experiments). *p < 0.05; **p < 0.01; ***p < 0.001; ns, no significance.

Given the essential impact of exosomal circCMTM3 derived from GSCs on the prognosis of GBM patients, we ulteriorly explored the contribution of cell-to-cell communication mediated by circCMTM3 in GDEs to the pathological progression of GBM. Due to the complex cellular and non-cellular components in the GBM microenvironment, GSCs and DGCs constitute the majority of the solid part of GBM^19^. Therefore, investigating the effects of exosomal circCMTM3 on the biological behavior of DGCs is of utmost importance. Based on our present inference, DGCs incubated with PKH26-labeled GDEs showed punctate red fluorescence signals within the cytoplasm, indicating uptake and internalization of GDEs by DGCs (Figure 2A). Furthermore, we performed knockdown and overexpression of circCMTM3 in GSCs and validated the changes through qPCR assays (Figure S2A). Simultaneously, the expression changes of exosomal circCMTM3 also followed the corresponding trends with the adjustment of circCMTM3 expression in GSCs (Figure S2B). Similarly, intervention with different types of GDEs resulted in the same changes of circCMTM3 in DGCs (Figure 2B). In conclusion, these findings collectively suggest that the dynamic changes of circCMTM3 in GSCs are perceived by DGCs through the transfer of exosomes.

### Exosomal circCMTM3 promotes DGCs to switch phenotype transition towards VM

To assess the direct influence of exosomal circCMTM3 on the malignant phenotype of DGCs, EdU, MTS, invasion, and migration assays were performed on DGCs under different interventions with GDEs. Results demonstrated that GDEs with circCMTM3 overexpression significantly enhanced the proliferation (Figure 2C-D, Figure S2C-D), invasion (Figure 2E-F), and migration (Figure 2G-H, Figure S2E-F) ability of DGCs. Conversely, downregulation of exosomal circCMTM3 notably weakened the malignant phenotype of DGCs.

GSCs are typically present in a perivascular niche, and angiogenesis is a crucial pathological feature of GBM, with microvascular-like structures containing abundant VM^20^. Based on the aforementioned theoretical foundation, we first performed Western blot analysis on DGCs from each treatment group to detect VM markers, including MMP2, VE-cadherin, and Vimentin^21^, ^22^. The results demonstrated that exosomal circCMTM3 intervention significantly increases the expression of VM-related markers, whereas downregulation of circCMTM3 leads to a marked reduction in the ability of DGCs to transform into VM structures (Figure 2I). Meanwhile, we also explored the tube-forming ability of DGCs under in vitro conditions. The results revealed that exosomal circCMTM3 had a vital function in promoting the occurrence of tubular structures (Figure 2J-L). Furthermore, transcriptional level analysis suggested a strong positive correlation between the expression of circCMTM3 and angiogenesis pathway genes (Figure S2G). Altogether, exosomal circCMTM3 might remodel the niche containing microvascular-rich structures by promoting the occurrence of VM.

### Identification of CNOT4 as the target of exosomal circCMTM3 in DGCs

**Figure 3:**
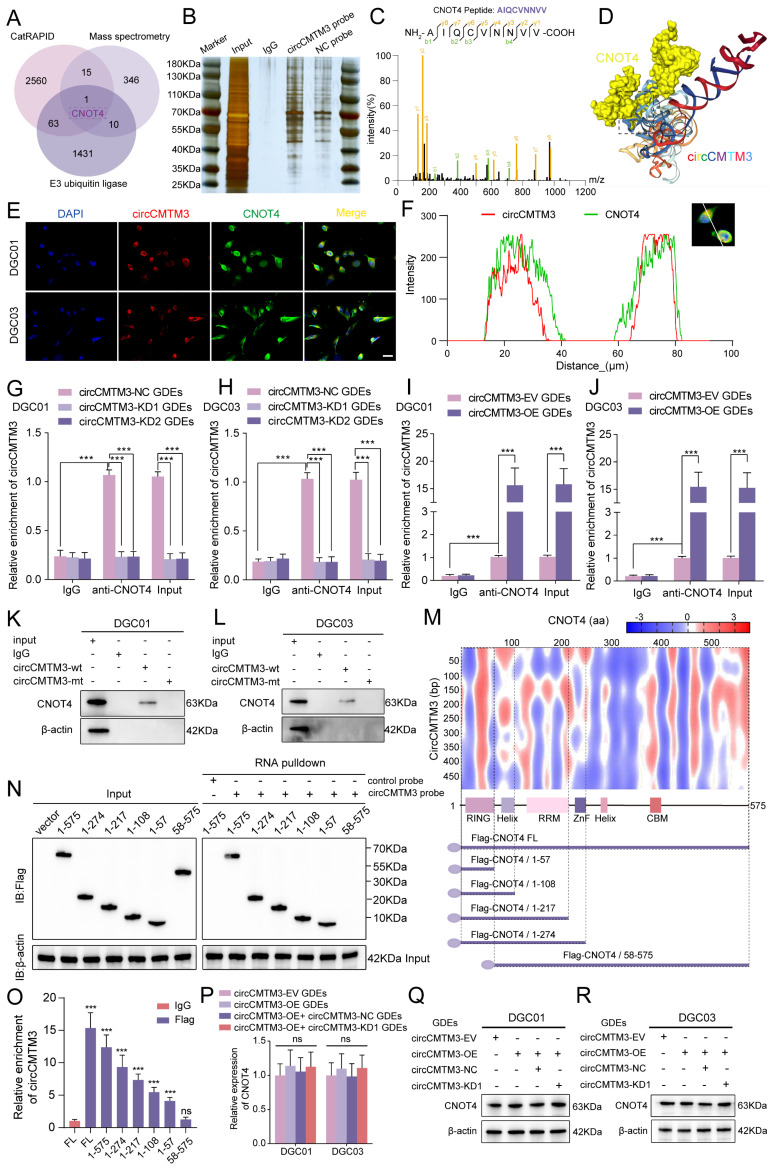
** Exosomal circCMTM3 targets and interacts with CNOT4 in DGCs. A** Venn chart of exosomal circCMTM3 downstream target screening. **B** Silver staining image of RNA pull-down assay with circCMTM3 and control probes in DGCs. **C** LC-MS/MS spectrum showing the CNOT peptides pulled down by circCMTM3 probe. **D** 3D schematic diagram predicting the interaction between circCMTM3 and CNOT4 via HDOCK. **E, F** Representative images of immunofluorescence staining (E) and line chart of fluorescence signal positioning analysis (F) showing the colocalization of circCMTM3 (red) and CNOT4 (green) in DGCs. Scale bar = 100 μm. **G-J** RIP assays showing anti-CNOT treatment leaded to exosomal circCMTM3 enrichment in DGCs by incubating with different groups of GDEs. **K, L** Western blot analysis after RNA pull-down assay to investigate the interaction between exosomal circCMTM3 and CNOT4 in DGCs. **M** Heatmap of RNA-protein interaction binding strength between circCMTM3 and CNOT4 via the CatRAPID algorithm (top) and the diagrams of domain structure of CNOT4 and Flag-tagged CNOT4 truncations (bottom). **N** Left, western blot analysis showing the expression of full length or CNOT4 truncations from DGCs transfected with the indicated vectors; Right, western blot analysis revealing the enriched CNOT4 truncations pulled down by circCMTM3 probe. **O** RIP assays displaying enrichment levels of circCMTM3 by anti-Flag in DGCs transfected with the truncated mutant vectors. **P** RT-qPCR assays showing the expression of CNOT4 in DGCs treated with different groups of GDEs. **Q, R** western blot assays revealing the expression of CNOT4 in DGCs after incubating with different groups of GDEs. Data are presented as means ± SD (three independent experiments). *p < 0.05; **p < 0.01; ***p < 0.001; ns, no significance.

Based on the specific function of exosomal circCMTM3 in promoting GBM VM formation, we further investigated the intracellular functions of circCMTM3 after its transportation into DGCs. Since the biological functions of circRNAs primarily depend on their cellular localization, cytoplasmic circRNAs exert their biological functions mainly through interactions with proteins^23^. Therefore, we used the constructed biotinylated circCMTM3 probe for RNA-pulldown assay combined with mass spectrometry analysis to explore the potential proteins that interact with circCMTM3 in DGCs (Table S6). Additionally, the CatRAPID database was used to predict the protein factors that bind to circCMTM3 (Table S7). Considering that the maintenance of protein homeostasis is a significant metabolic event controlling the proliferation and progression of malignant tumors, and protein homeostasis is often subjected to dysregulated regulation by the Ubiquitin-Proteasome System (UPS). As a pivotal molecule in the UPS, the integrity of ubiquitin ligases is closely related to the malignant transformation of tumors^24^. Therefore, we performed an intersection analysis of the CatRAPID prediction results, mass spectrometry analysis results, and all E3 ubiquitin ligases (Table S8), identifying CNOT4 as the sole E3 ubiquitin ligase that could potentially interact with circCMTM3 (Figure 3A-C). Furthermore, the HDOCK structural alignment tool simulated the 3D model of the complex formed by CNOT4 and circCMTM3, indicating that the main binding site of CNOT4 with circCMTM3 is located in its N-terminal domain (Figure 3D). To validate the above conclusions, we conducted fluorescence co-localization assay of CNOT4 and circCMTM3, and as hypothesized, both co-localized in the cytoplasm of DGCs (Figure 3E-F). Next, RIP and RNA-pulldown assays were conducted to confirm the interaction between CNOT4 and circCMTM3. The results of RIP assays demonstrated that after anti-CNOT4 treatment a higher enrichment of circCMTM3 was detected compared to the IgG group. Moreover, DGCs incubated with different types of GDEs showed a higher enrichment of circCMTM3 accompanied by overexpression of exosomal circCMTM3, while the enrichment of circCMTM3 decreased observably with the knockdown of exosomal circCMTM3 (Figure 3G-J). Additionally, the results of RNA-pulldown assays demonstrated that the wild-type circCMTM3 probe pulled down CNOT4, whereas the mutant probe did not in DGCs (Figure 3K-L).

### CircCMTM3 precisely binds to the N-terminal RING domain of CNOT4

To investigate the precise binding site of CNOT4 and circCMTM3, a series of truncated mutant constructs of Flag-tagged CNOT4 were designed and cloned for RNA-pulldown and RIP assays based on the predicted binding strength from CatRAPID and the arrangement and positions of Human CNOT4 domains (Figure 3M)^25^. Consistent with the simulation results of the HDOCK tool, the biotinylated circCMTM3 probe only pulled down the N-terminal domain of CNOT4, such as △1-57aa, △1-108aa, △1-217aa, and △1-274aa, while △58-575aa could not be pulled down by the RNA probe intriguingly (Figure 3N). Similarly, RIP assays also revealed that the N-terminal truncated mutant containing the structure of 1-57aa exhibited the enrichment effect of circCMTM3, while △58-575aa did not, compared to the IgG negative control (Figure 3O). Therefore, the RING domain of CNOT4, which is also a domain with E3 ligase catalytic activity, is necessary for binding to circCMTM3. In addition, we detected the mRNA and protein levels of CNOT4 in DGCs treated with different types of GDEs, and discovered that the upregulation or downregulation of exosomal circCMTM3 did not affect the transcription and translation levels of CNOT4 (Figure 3P-R). In conclusion, the above research results confirm that the interaction between CNOT4 and circCMTM3 occurs in the N-terminal RING domain, and this interaction does not affect the expression of CNOT4.

### CNOT4 induces ubiquitination of STAT5A/B and expedites their degradation

**Figure 4:**
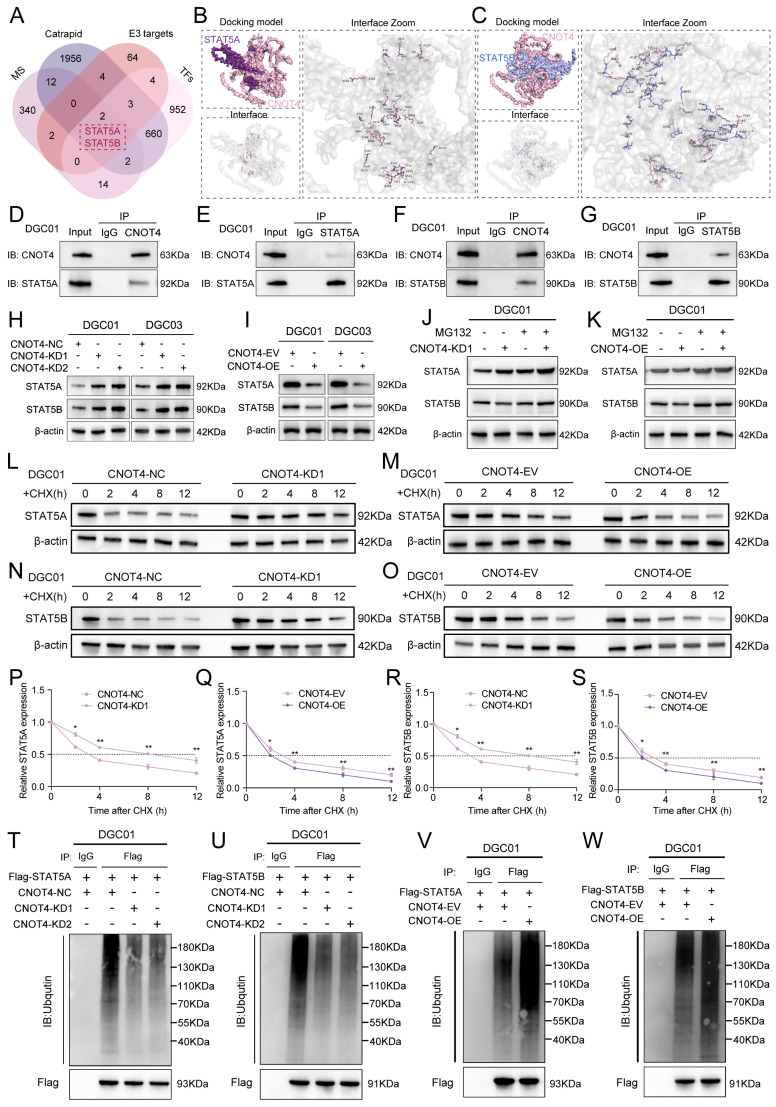
** CNOT4 induces ubiquitination and degradation of STAT5A/B in DGCs. A** Venn diagram showing integrated CatRAPID, mass spectrometry, TFs and UbiBrowser screening results. **B, C** Surface diagram of the docking model and interfacing residues between CNOT4 and STAT5A proteins (B) as well as CNOT4 and STAT5B (C) by GRAMM-X.** D-G** Western blotting analysis after Co-IP assays to assess the interaction of CNOT4 and STAT5A (D, E) as well as CNOT4 and STAT5B (F, G).** H, I** Western blotting analysis to confirm STAT5A and STAT5B expression after CNOT4 downregulation (H) or overexpression (I) in DGCs.** J, K** Western blotting assays showing STAT5A and STAT5B expression in CNOT4-silenced DGC01 (J) or overexpressed DGC01(K) treated with or without MG-132 (50 μM) for 6 h.** L-O** Western blotting assays showing STAT5A and STAT5B expression at different time nodes in CNOT4-silenced DGC01 (L, M) or overexpressed DGC01(N, O) by treating with CHX (50 μg/ml).** P-S** Quantitative analysis revealing half-life time(t_1/2_) of STAT5A (P, Q) and STAT5B (R, S) expression by regulating CNOT4 in DGCs. **T-W** Ubiquitination assays showing the STAT5A and STAT5B ubiquitination levels followed by CNOT4 silencing (T, U) or overexpressed (V, W) in DGC01with MG132 treatment (50 μM) for 6 h. Data are presented as means ± SD (three independent experiments). *p < 0.05; **p < 0.01; ***p < 0.001; ns, no significance.

The targeted effect of exosomal circCMTM3 on CNOT4 in DGCs may be achieved by blocking its ubiquitin ligase activity. Therefore, we used UbiBrowser 2.0 to predict the proteins targeted by CNOT4 (Table S9). Considering that transcription factors is crucial in regulating tumor vasculogenesis, we integrated and screened the obtained mass spectrometry data, CatRAPID prediction results, the transcription factors set acquired from the Cistrome Data Browser (Table S10), and CNOT4 targeting proteins. We identified STAT5A and STAT5B as the most likely downstream factors involved in the regulation of VM formation by exosomal circCMTM3 (Figure 4A). Molecular docking was performed utilizing the GRAMM-X tool to describe proteins interaction. Molecular docking analysis revealed that CNOT4 interacts with both STAT5A and STAT5B respectively through sharing structural motif for direct contact. Visual prediction illustration generated from GRAMM Docking exhibited protein binding interface between CNOT4 and STAT5A/B (Figure 4B-C). Based on qualitative analysis of molecular binding sites, we further explored the regulatory mechanism of CNOT4 on the expression of STAT5A and STAT5B in DGCs. First, The Co-IP assay results revealed that CNOT4 can interact with STAT5A and STAT5B, forming protein complexes individually (Figure 4D-G). Furthermore, western blot demonstrated significant alterations in the protein expression levels of STAT5A and STAT5B accompanied by CNOT4 overexpression and knockdown. The downregulation of CNOT4 resulted in a prominent increase in the levels of STAT5A and STAT5B, whereas the overexpression of CNOT4 led to their subsequent downregulation (Figure 4H-I). However, no significant changes in mRNA levels of STAT5A and STAT5B were detected under conditions of CNOT4 gene manipulation (Figure S3A-D). Pertinently, the degradation of STAT5A and STAT5B induced by CNOT4 overexpression was effectively rescued through treatment with the proteasome inhibitor MG132. Moreover, the upregulation of STAT5A and STAT5B induced by CNOT4 knockdown was further augmented by MG132 treatment, leading to their enhanced accumulation (Figure 4J-K). Additionally, CHX pulse-chase assays confirmed a significant extension in the half-life of STAT5A and STAT5B when subjected to CNOT4 silencing conditions. Conversely, CNOT4 overexpression led to more rapid degradation of STAT5A and STAT5B compared to the control group (Figure 4L-S). These findings suggest that CNOT4 may regulate the expression of STAT5A and STAT5B depending on its function as an E3 ubiquitin ligase. To validate this hypothesis, ubiquitination analysis of STAT5A and STAT5B was performed, and the results showed that CNOT4 silencing notably decreased ubiquitination levels of STAT5A and STAT5B, while overexpression of CNOT4 ameliorated loss of ubiquitination (Figure 4T-W). Overall, CNOT4, as an E3 ubiquitin ligase, can regulate the post-transcriptional expression levels of STAT5A and STAT5B through catalyzing their ubiquitination modification in DGCs.

### Exosomal circCMTM3 prevents ubiquitinational degradation of STAT5A/B though competitively binding to CNOT4

**Figure 5:**
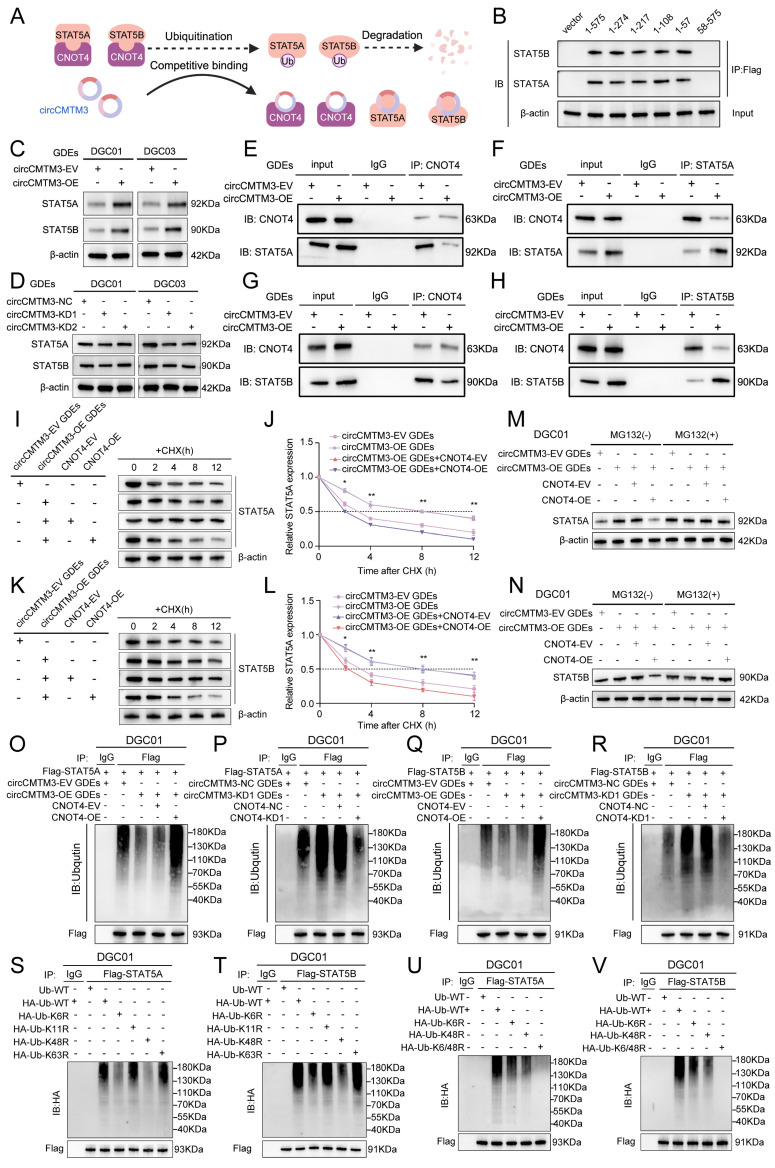
** Exosomal circCMTM3 competitively binds to CNOT4 and prevents from STAT5A/B degradation in DGCs. A** Schematic diagram of the regulatory mode reflecting circCMTM3 impacting on STAT5A/B expression.** B** Co-IP assay displaying the binding domain of CNOT4 responsible for its interaction with STAT5A/B in DGCs transfected with the truncated mutant vectors.** C, D** Western blotting assays showing STAT5A and STAT5B expression in DGCs with treatment of different groups of GDEs.** E-H** Co-IP assays illustrating interaction efficiency of CNOT4 and STAT5A (E, F) as well as CNOT4 and STAT5B (G, H) in DGCs under the condition of exosomal circCMTM3 overexpression. **I-L** Western blotting analysis showing STAT5A and STAT5B expression in CNOT4-overexpressed DGCs with treatment by GDEs containing upregulated circCMTM3 and CHX (50 μg/ml) (I, K), meanwhile, quantitative analysis on STAT5A and STAT5B expression half-life time (t_1/2_) reflecting degradation rates (J, L).** M, N** Western blotting assays showing STAT5A (M) and STAT5B (N) expression in CNOT4-overexpressed DGC01 treated with or without MG-132 (50 μM) after incubating with circCMTM3-upregulated GDEs.** O-R** Ubiquitination assays showing the STAT5A and STAT5B ubiquitination levels in DGC01 with MG132 treatment (50 μM) followed by CNOT4 overexpression combined with circCMTM3-upregulated GDEs treatment (O, Q) or CNOT4 and exsomal circCMTM3 silenced simultaneously (P, R). **S, T** In vivo ubiquitination assays of polyubiquitin chains of STAT5A (S) and STAT5B (T) in DGCs transfected with mutant ubiquitin plasmids at the K6, K11, K48, and K63 sites.** U, V** Ubiquitination assays showing polyubiquitin chains assembly of STAT5A (U) and STAT5B (V) in DGCs transfected with wild-type or K6R, K48R and K6/48R-mutant ubiquitin plasmids. Data are presented as means ± SD (three independent experiments). *p < 0.05; **p < 0.01; ***p < 0.001; ns, no significance.

Based on the prediction results from CatRAPID, exosomal circCMTM3 can interact with both STAT5A and STAT5B in DGCs (Figure S3E-F). Furthermore, circCMTM3 functions as a protector against the ubiquitination degradation of targeting proteins by obstructing the E3 ubiquitin ligase domain of CNOT4. Therefore, our hypothesis posits that exosomal circCMTM3 may uphold the stability of STAT5A and STAT5B proteins through competitive binding with CNOT4 and interaction with STAT5A/STAT5B (Figure 5A). To validate this assumption, we first investigated the binding sites between CNOT4 and STAT5A/STAT5B. As expected, we observed that STAT5A/STAT5B primarily interacted with the N-terminal domain of CNOT4, including the RING domain (Figure 5B). In addition, the overexpression of exosomal circCMTM3 significantly upregulated the protein levels of STAT5A/STAT5B, while the suppression of exosomal circCMTM3 resulted in a remarkable decrease of STAT5A/STAT5B expression (Figure 5C-D). There were no statistical differences in the transcription levels of STAT5A/STAT5B in response to alterations in exosomal circCMTM3 expression in DGCs, confirmed by qPCR assay (Figure S3G-J).

Furthermore, a series of Co-IP assays were conducted to validate whether circCMTM3 increases the expression of STAT5A/STAT5B by competitively binding with CNOT4 under conditions of exosomal circCMTM3 knockdown or overexpression. We observed that the overexpressed exosomal circCMTM3 hindered the interaction between CNOT4 and STAT5A/STAT5B, while simultaneously elevating their expression levels (Figure 5E-H). Conversely, silencing exosomal circCMTM3 enhanced the binding of CNOT4 with STAT5A/STAT5B, leading to bulk degradation of STAT5A/STAT5B (Figure S3K-N). Moreover, CHX pulse-chase assays indicated that prolonged half-life conferred on STAT5A/STAT5B due to overexpression of exosomal circCMTM3 was significantly shortened upon upregulation of CNOT4 (Figure 5I-L). Instead, silencing CNOT4 considerably delayed the shortening in half-life caused by knockdown of exosomal circCMTM3 (Figure S3O-R). Similarly, upregulation of CNOT4 attenuated the accumulation of STAT5A/STAT5B induced by Intervention of increased exosomal circCMTM3, which could be completely blocked by treatment with MG132 (Figure 5M-N). Correspondingly, downregulation of CNOT4 rescued the degradation of STAT5A/STAT5B caused by silencing exosomal circCMTM3 and stabilized high levels of STAT5A/STAT5B expression under MG132 treatment (Figure S3S-T). Furthermore, ubiquitination assays revealed that the aggregation of ubiquitin chains linked to STAT5A/STAT5B could be alleviated by the upregulation of exosomal circCMTM3, whereas the complementation of CNOT4 observably enhanced the polyubiquitination of STAT5A/STAT5B. On the contrary, the increased ubiquitination modification of STAT5A/STAT5B caused by the silencing of exosomal circCMTM3 was abolished in the absence of CNOT4 (Figure 5O-R). In conclusion, circCMTM3 derived from GDEs can protect STAT5A/STAT5B from degradation by competitively binding with CNOT4 and blocking its E3 ubiquitin ligase catalytic activity, thus enhancing the stability of STAT5A/STAT5B expression in DGCs.

### CNOT4 mediates dysfunctional degradation of STAT5A/B by catalyzing the assembly of K6 and K48 polyubiquitin chains

Previous studies have reported that different types of E3 ubiquitin ligases play a crucial role in determining the fate of downstream target proteins through assembling various non-canonical polyubiquitin chains. Non-canonical polyubiquitin chains formed via K6, K11, K48, and K63 linkages are widely associated with protein degradation and malignant tumor progression^26^. To validate CNOT4's impact on the ubiquitination modification pattern of downstream target proteins, we generated a series of vectors carrying point mutations at ubiquitin lysine residues including Lys6, Lys 11, Lys 48, and Lys 63, along with a HA tag, for subsequent in vivo ubiquitination studies. Our results indicated that introducing a K48R mutation significantly reduced polyubiquitin chain formation on STAT5A/STAT5B, while the K6R mutation had a slight attenuating effect on them (Figure 5S-T). Furthermore, we constructed a vector harboring simultaneous mutations at Lys6 and Lys48 (K6/48R) which were then transfected into DGCs. As expected, under these conditions where both K6 and K48 were mutated, nearly all polyubiquitin chain modifications associated with STAT5A/STAT5B were abolished (Figure 5U-V). Collectively, our findings suggest that CNOT4 promotes non-canonical polyubiquitination modifications specifically through K48- and K6-linkages on STAT5A/STAT5B leading to its degradation.

### Exosomal circCMTM3 acts as a molecular scaffold causing differential phosphorylation levels of STAT5A and STAT5B

**Figure 6:**
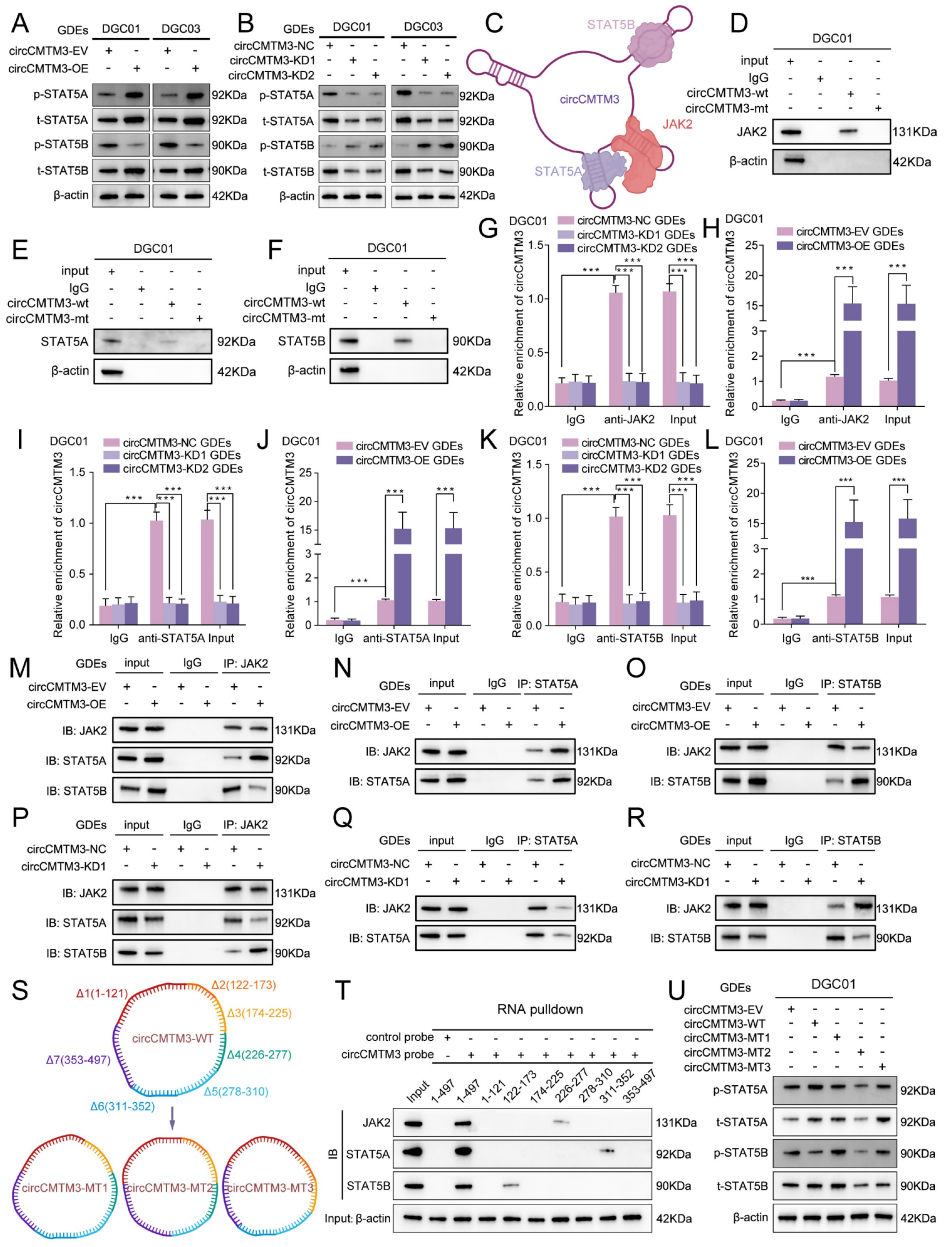
** Exosomal circCMTM3 serves as a molecular scaffold activating JAK2/STAT5A pathway in DGCs. A, B** Western blotting analysis showing protein levels of p-STAT5A (S726), total STAT5A, p-STAT5B (S731) and total STAT5B in DGCs after incubating with circCMTM3-upregulated or silencing GDEs. **C** Schematic diagram illustrating circCMTM3 as a molecular scaffold leading to rearrangement of the spatial positions of JAK2, STAT5A, and STAT5B. **D-F** RNA pull-down assay to confirm the interaction between exosomal circCMTM3 and JAK2 (D), STAT5A (E), and STAT5B (F) respectively in DGC01. **G-L** RIP assays showing anti-JAK2 (G, H), anti-STAT5A (I, J) and anti-STAT5B (K, L) treatment leaded to exosomal circCMTM3 enrichment in DGC01 by incubating with different groups of GDEs.** M-R** Co-IP assays displaying interaction efficiency of JAK2 and STAT5A as well as JAK2 and STAT5B in DGCs under the condition of exosomal circCMTM3 overexpression (M-O) and knockdown (P-R).** S** Illustration of functional fragments of circCMTM3 (upper panel) and corresponding deletion mutants (down panel) predicted and divided by the CatRAPID tool. **T** Western blot analysis revealing the affinity between different circCMTM3 fragment probe and STAT5A, STAT5B and JAK2 proteins via RNA pulled down assays. **U** Western blotting analysis illustrating protein levels of p-STAT5A (S726), total STAT5A, p-STAT5B (S731) and total STAT5B in DGCs treated by different mutant circCMTM3-riched GDEs. Data are presented as means ± SD (three independent experiments). *p < 0.05; **p < 0.01; ***p < 0.001; ns, no significance.

STAT5A/B, as members of the STATs family, possessed phosphorylated transactivation domains closely linked to their transcriptional activity, where the phosphorylation modification of the STAT5A-S726 and STAT5B-S731 sites commonly used to assess the degree of transcriptional activation^27^. The functional role of circCMTM3 in STAT5A/STAT5B may extend beyond protecting them from ubiquitination degradation, and whether it also regulates the phosphorylation levels of STAT5A/STAT5B is worth exploring. Therefore, we examined the phosphorylation levels of STAT5A-S726 and STAT5B-S731 under conditions of exosomal circCMTM3 overexpression and silencing. Intriguingly, the overexpression of exosomal circCMTM3 dramatically increased p-STAT5A level, while markedly decreasing p-STAT5B protein level. In contrast, silencing exosomal circCMTM3 led to a significant decrease in p-STAT5A level but an increase in p-STAT5B levels (Figure 6A-B). Given the abundant cytoplasmic expression of circCMTM3 and its potent protein-binding function, we hypothesize that circCMTM3 may act as a protein scaffold to influence the classical activation pathway of STAT5A/STAT5B. The upstream kinase JAK2 is responsible for mediating the phosphorylation activation process of both STAT5 proteins. Examination of the mass spectrometry analysis of circCMTM3-binding candidates revealed the presence of JAK2 and STAT5A/B in the list (Figure S4A-C).

Combining with the detailed binding sites predicted by CatRAPID for the interaction between circCMTM3 and STAT5A, STAT5B, and JAK2, it is suggested that distinct stem-loop domains of circCMTM3 may serve as protein scaffolds to reset the spatial localization of STAT5A, STAT5B, and JAK2, thereby influencing the phosphorylation levels of STAT5A and STAT5B (Figure 6C). To elucidate our hypothesis accurately, we initially performed RNA-pulldown assays, and observed that the wild-type circCMTM3 probe could effectively pull down STAT5A, STAT5B, and JAK2, while the mutant probe could not (Figure 6D-F, Figure S4D-F). Additionally, RIP assays demonstrated that exosomal circCMTM3 could be specifically enriched by STAT5A, STAT5B, and JAK2 individually in DGCs (Figure 6G-L, Figure S4G-L).

### The binding preference of circCMTM3 fragments leads to rearrangement of the spatial positions of JAK2, STAT5A, and STAT5B

Co-IP assay was conducted to verify whether circCMTM3 could function as a scaffold to enhance the interaction between JAK2 and STAT5A while impairing the combination of STAT5B and JAK2. As expected, the overexpression of exosomal circCMTM3 enhanced binding between JAK2 and STAT5A while weakening the interaction between JAK2 and STAT5B (Figure 6M-O). Inversely, silencing exosomal circCMTM3 strengthened binding between JAK2 and STAT5B, while the binding effect of JAK2 to STAT5A was weakened (Figure 6P-R). Due to the apparent specificity of the binding sites identified for interactions between circCMTM3 and STAT5A, STAT5B, or JAK2, RNA pulldown assays were performed using biotinylated probes containing different structural fragments of circCMTM3, which is based on the detailed division of its functional sequences predicted by the CatRAPID tool (Figure 6S, upper panel). The results confirmed that STAT5A preferred binding to the 311-353 region, while STAT5B had higher affinity towards the 122-173 sequence, and JAK2 exhibited a more significant interaction with the 226-277 segment of circCMTM3 (Figure 6T). Additionally, deletion mutants of the aforementioned protein-binding fragments mentioned were expressed in DGCs to assess the overall expression and phosphorylation levels of STAT5A and STAT5B (Figure 6S, down panel). The findings revealed that circCMTM3-MT1, lacking the 122-173 fragment, failed to restrain the spatial positioning of STAT5B, resulting in competition between STAT5A and STAT5B, which partially increased the phosphorylation modification of STAT5B. CircCMTM3-MT2, due to the loss of its binding capacity to STAT5A, led to a conspicuous decrease in p-STAT5A, while having minimal impact on p-STAT5B. CircCMTM3-MT3, lacking the auxiliary scaffold effect on JAK2, resulted in equal opportunities for phosphorylation modification of both STAT5A and STAT5B compared to the wild-type (Figure 6U). Altogether, exosomal circCMTM3 can rearrange the spatial positioning of JAK2, STAT5A, and STAT5B within DGCs through its protein scaffold function, facilitating the phosphorylation of STAT5A while impeding that of STAT5B.

### Exosomal circCMTM3 facilitates VM formation in vitro via boosting p-STAT5A expression

A series of in vitro cell phenotype assays were conducted to investigate the function of p-STAT5A in promoting VM in GBM. Firstly, the results of immunofluorescence assays demonstrated that exosomal circCMTM3 overexpression not only upregulated the expression of p-STAT5A but also enhanced its nuclear translocation (Figure S6A). A small molecule inhibitor, Stafia-1^28^, has been reported to possess high selectivity for STAT5A and can specifically inhibit the transcriptional activity. Subsequently, MTS, EdU, invasion, migration, and tube formation assays all provided evidence that the enhancement of proliferation (Figure S5A-D), invasion (Figure S5E-F), migration (Figure S5G-H), and tube-forming abilities (Figure S5I-K) in DGCs due to exosomal circCMTM3 overexpression, could be obviously eliminated by Stafia-1. To conclude, the facilitation of VM by exosomal circCMTM3 is achieved in GBM by motivating the expression and nuclear translocation of p-STAT5A.

### STAT5A transcriptionally upregulates the expression of the pro-vasculogenic factor CHI3L2

**Figure 7:**
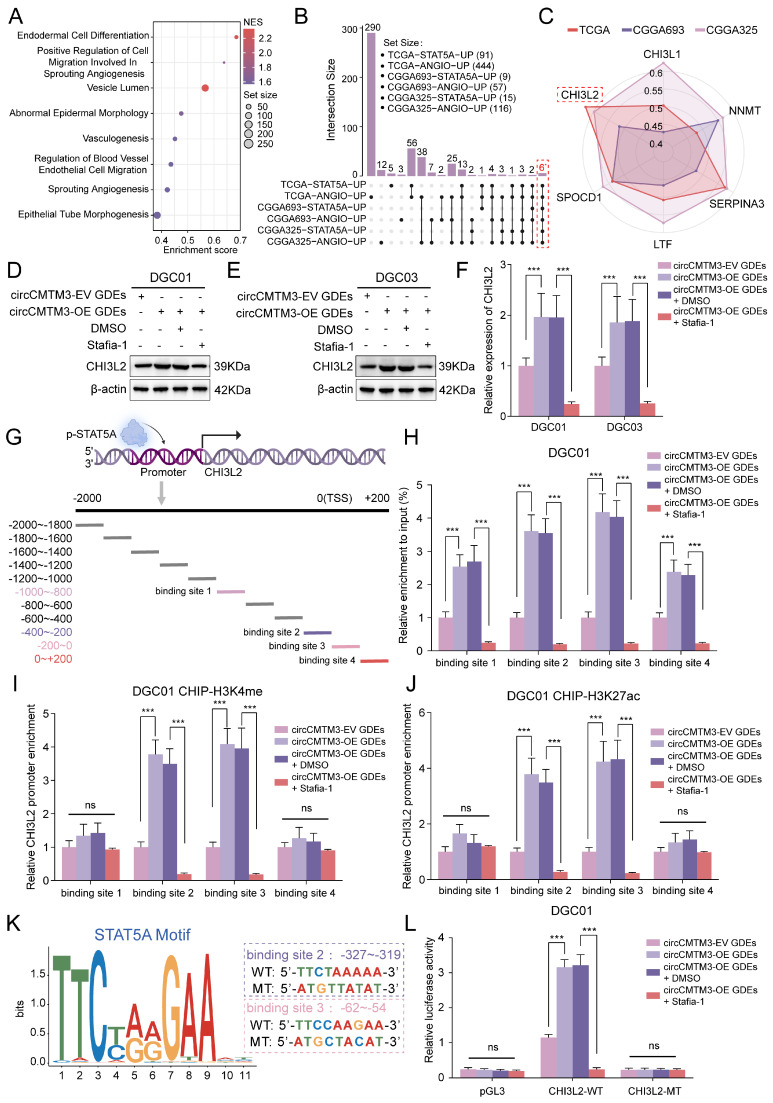
** STAT5A transcriptionally upregulates the expression of the pro-vasculogenic factor CHI3L2. A** GSEA based on bulk RNA-seq data of TCGA-glioma with STAT5A expressional difference. **B** Upset plot illustrating the number of genes obtained by differential analysis based on STAT5A expression and GSVA score of angiogenesis pathway in TCGA, CGGA325 and CGGA693 glioma samples. **C** Six candidate genes were screened from the overlap of the TCGA-STAT5A-UP, TCGA-ANGIO-UP, CGGA693-STAT5A-UP, CGGA693-ANGIO-UP, CGGA325-STAT5A-UP and CGGA325-ANGIO-UP data. The different polygons represent the correlation of candidates and STAT5A expression levels in TCGA, CGGA325 and CGGA693 respectively. **D, E** Western blotting assays showing CHI3L2 expression in DGC01 and DGC03 treated with or without Stafia-1 (20 μM) after incubating with circCMTM3-upregulated GDEs. **F** RT-qPCR assays displaying the mRNA expression of CHI3L2 in DGCs intervened with or without Stafia-1 (20 μM) after treatment with circCMTM3-overexpressed GDEs. **G** Schematic diagram showing the promoter region of CHI3L2 is divided into 11 parts for ChIP assays to validate the transcriptional activity of STAT5A. **H** Quantitative analysis of ChIP assay indicating the STAT5A binding regions in the CHI3L2 promoters under the condition of exosomal circCMTM3 overexpression with or without Stafia-1intervention. **I, J** ChIP-qPCR detecting H3K4me (I) and H3K27ac (J) enrichment in the CHI3L2 promoters in DGC01 with different group GDEs treatment and Stafia-1or DMSO intervention. **K** Schematic representation of the binding sites for STAT5A in the CHI3L2 promoters obtained from HOCOMOCO (left). Sequences of the wild-type (WT) and mutated (MT) binding sites in the promoter regions used in the luciferase reporter plasmids (right). **L** Dual luciferase reporter assay indicating that exosomal circCMTM3 increased the activity of the wild-type CHI3L2 promoter but had no effect on the activity of the mutated binding sites. Stafia-1 presenting transcriptional inhibition in DGC01. Data are presented as means ± SD (three independent experiments). *p < 0.05; **p < 0.01; ***p < 0.001; ns, no significance.

Considering the classical transcription factor activity of p-STAT5A, we aimed to explore the intrinsic regulatory mechanisms underlying GBM neovascularization mediated by STAT5A. Gene Set Enrichment Analysis (GSEA) was initially performed which revealed enrichment of multiple signaling pathways related to vasculogenesis in glioma samples with high STAT5A expression from TCGA dataset (Figure 7A). In addition, differential gene analysis between STAT5A high and low expression groups was conducted using TCGA-Glioma, CGGA693, and CGGA325 datasets containing extensive glioma tissue bulk RNA-sequencing data. Meanwhile, each glioma sample was evaluated for angiogenesis pathway score using Gene Set Variation Analysis (GSVA), then differential analysis between high and low score groups identified genes strongly associated with neovascularization. Ultimately, we identified 6 candidate genes that overlapped in the differential gene lists across all datasets (Figure 7B). Based on the correlation levels of these 6 candidates with STAT5A in different glioma datasets and comprehensive evaluation, we focused on CHI3L2 and explored further (Figure 7C, Figure S6B).

To confirm whether the expression level of CHI3L2 is regulated by nuclear translocation of p-STAT5A, WB and qPCR assays were performed. We observed that the overexpression of exosomal circCMTM3 noticeably upregulated the transcriptional and translational expression of CHI3L2, while Stafia-1 effectively restricted the circCMTM3-induced high expression of CHI3L2 (Figure 7D-F). To validate the transcriptional activation of CHI3L2 by p-STAT5A, we designed 11 primer pairs covering all possible binding regions of p-STAT5A in the promoter region of CHI3L2 (Figure 7G). ChIP-qPCR assay displayed that p-STAT5A occupied 4 sites within the CHI3L2 promoter region (Figure S6C). Furthermore, upregulated circCMTM3 derived from GDEs led to abundant enrichment of the aforementioned 4 sites in the presence of anti-p-STAT5A. Pertinently, Stafia-1 significantly inhibited the occupancy of p-STAT5A within the CHI3L2 promoter region in DGEs (Figure 7H, Figure S6D). Additionally, we confirmed that p-STAT5A could increase the enrichment of chromatin activation markers, H3K4me and H3K27ac, at binding sites 2 and 3 (Figure 7I-J, Figure S6E-F). AnimalTFDB database was then utilized to predict the sequence information of the CHI3L2 promoter region bound by p-STAT5A (Table S11), and corresponding sequences in binding sites 2 and 3 were mutated for subsequent luciferase reporter gene assays (Figure 7K). Results indicated that exosomal circCMTM3 enhanced the luciferase activity of the pGL3-CHI3L2-WT vector by upregulating p-STAT5A, while Stafia-1 completely blocked activating effects exerted by p-STAT5A on this vector in DGCs (Figure 7L, Figure S6G). To summarize, p-STAT5A promotes the formation of VM by transcriptionally upregulating the expression of CHI3L2.

### SRSF1 maintains the constant expression of exosomal circCMTM3 in DGCs

**Figure 8:**
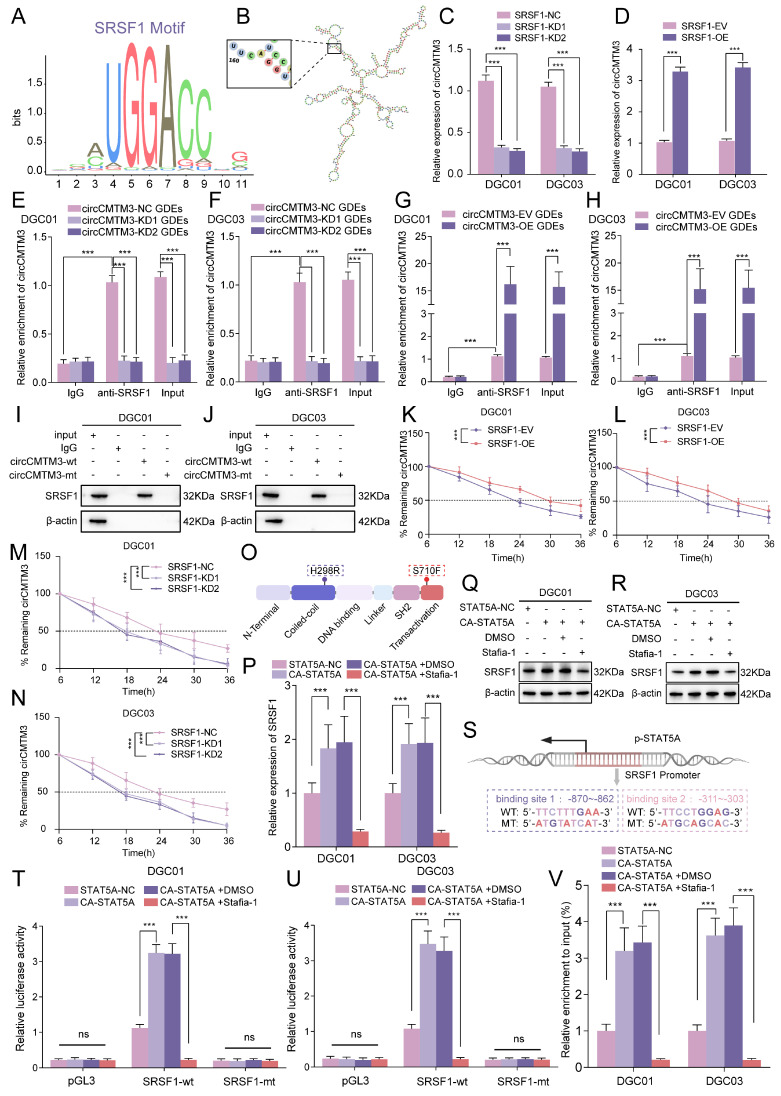
** SRSF1 maintains the stability of exosomal circCMTM3 and is transcriptionally upregulated by STAT5A in DGCs. A** SRSF1 motif sequence identified by RBP suite database. **B** Schematic representation illustrating RNA secondary stem-loop structures of circCMTM3 predicted by cRNAsp12. The characteristic binding site sequence interacted with SRSF1 is exhibited within the enlarged box on the left of the panel. **C, D** RT-qPCR assays displaying the RNA expression of exosomal circCMTM3 in DGCs intervened with GDEs in SRSF1-silencing (C) and overexpressed (D) DGCs. **E-H** RIP assays showing anti-SRSF1 treatment caused exosomal circCMTM3 enrichment in DGC01 (E, G) and DGC03 (F, H) by treatment with different groups of GDEs. **I, J** RNA pull-down assay to validate the interaction between exosomal circCMTM3 and SRSF1 in DGC01 (I) and DGC03 (J). **K-N** RNA dynamic assays showing the half-life of exosomal circCMTM3 in SRSF1- overexpressed (K, L) or silencing (M, N) DGCs followed by actinomycin D treatment. **O** Schematic illustration displaying CA-STAT5A domain structure of CA-STAT5A carrying mutations at two amino acids sites. **P** RT-qPCR assays showing the mRNA expression of SRSF1 in DGCs via CA-STAT5A overexpression and intervention by Stafia-1 or DMSO. **Q, R** Western blotting assays showing SRSF1 expression in DGC01(q) and DGC03 (r) treated with or without Stafia-1 after CA-STAT5A overexpression. **S** Schematic representation of the binding sites for STAT5A in the SRSF1 promoters and matched mutant sequences for Dual-luciferase reporter assays. **T, U** The Dual-luciferase reporter assays revealing the luciferase promoter activities of SRSF1 in DGC01 (T) and DGC03 (U) with or without CA-STAT5A overexpression and treatment by Stafia-1 or DMSO.** V,** The ChIP qPCR showing the enrichment difference of SRSF1 promoter sequence via anti-STAT5A treatment in DGCs with CA-STAT5A overexpression and treatment by Stafia-1 or not. Data are presented as means ± SD (three independent experiments). *p < 0.05; **p < 0.01; ***p < 0.001; ns, no significance.

Exosomal circCMTM3 can enter DGCs and maintain stable expression without degradation, potentially due to the protective role of RNA-binding proteins(RBPs)^29^. RBPs have been reported to participate in various biological processes related to circRNAs, including splicing, synthesis, localization, and stability. Therefore, we utilized the RBP suite database to predict and score RBPs with circCMTM3-binding potential (Table S12). SRSF1 emerges as the highest-scoring RBP, indicating a strong likelihood of binding to circCMTM3. The characteristic binding motif of SRSF1 is located within the secondary stem-loop structure of circCMTM3 that corresponds to the predicted binding segment identified by RBP suite (Figure 8A-B). Knockdown or overexpression of SRSF1 were performed in DGCs followed by incubation with GDEs carrying circCMTM3, then the quantitative analysis of exosomal circCMTM3 were evaluated using qPCR assays in DGCs. The results demonstrated that upregulation of SRSF1 enhanced the enrichment of exosomal circCMTM3, while silencing SRSF1 led to a decrease in the content of exosomal circCMTM3 (Figure 8C-D). To further confirm the functional interaction between circCMTM3 and SRSF1, RIP and RNA-pulldown assays were conducted on DGCs treated with different types of GDEs. The results from RIP assays showed that exosomal circCMTM3 was enriched by anti-SRSF1 treatment (Figure 8E-H). Furthermore, RNA-pulldown assays demonstrated that the wild-type circCMTM3 probe exhibited high affinity for SRSF1, while the mutant probe did not in DGCs (Figure 8I-J). To further validate the contribution of SRSF1 towards maintaining stability of exosomal circCMTM3 upon entry into DGCs, we determined the half-life of circCMTM3 from GDEs using RNA expression dynamics under manipulating SRSF1 expression levels. Consistent with expectations, upregulation of SRSF1 significantly delayed the decay of circCMTM3, while knockdown of SRSF1 accelerated circCMTM3 degradation (Figure 8K-N). In summary, SRSF1 enhances the stability of exosomal circCMTM3 in DGCs by exerting its RNA-binding function.

### STAT5A transcriptionally activates SRSF1 and triggers a positive feedback loop in DGCs

The initiation and progression of malignant tumors are not solely attributed to single-gene abnormalities, but rather a result of the cumulative effects of multiple gene anomalies acting synergistically. Therefore, to better elucidate how SRSF1 selectively enhances the stability of exosomal circCMTM3, further exploration was conducted on whether STAT5A has potential transcriptional activation effects on SRSF1. Interestingly, analysis and prediction results from the AnimalTFDB database exhibited the presence of STAT5A binding sites in the promoter region of SRSF1 (Table S11). Previous studies have reported that STAT5A has a constitutively activated mutation with changes at two positions, H298R and S710F (Figure 8O)^30^. Under the condition of overexpressing this mutant variant, CA-STAT5A, the expression of SRSF1 was noticeably upregulated at both mRNA and protein levels. Homoplastically, Stafia-1 completely suppressed the transcriptional activation effect of CA-STAT5A on SRSF1 (Figure 8P-R). Based on the predicted STAT5A binding sites in the SRSF1 promoter region, nucleic acid sequences carrying point mutations in the promoter regions were designed and cloned into pGL3 plasmids (Figure 8S). Subsequently, luciferase reporter gene assays were performed, and the results displayed that knock-in of CA-STAT5A obviously enhanced luciferase activity, while Stafia-1 completely suppressed the activation effect of CA-STAT5A on luciferase (Figure 8T-U). Similarly, ChIP assays revealed a conspicuous enrichment of the SRSF1 promoter region fragments caused by the expression of CA-STAT5A, and Stafia-1 completely suppressed the DNA-binding function of CA-STAT5A (Figure 8V). In summary, STAT5A triggers a positive feedback loop consisting of SRSF1, exosomal circCMTM3 and STAT5A in DGCs by upregulating the expression of SRSF1, while maintaining VM phenotype.

### Exosomal circCMTM3 accelerates GBM malignant progression via inducing VM formation in vivo

**Figure 9:**
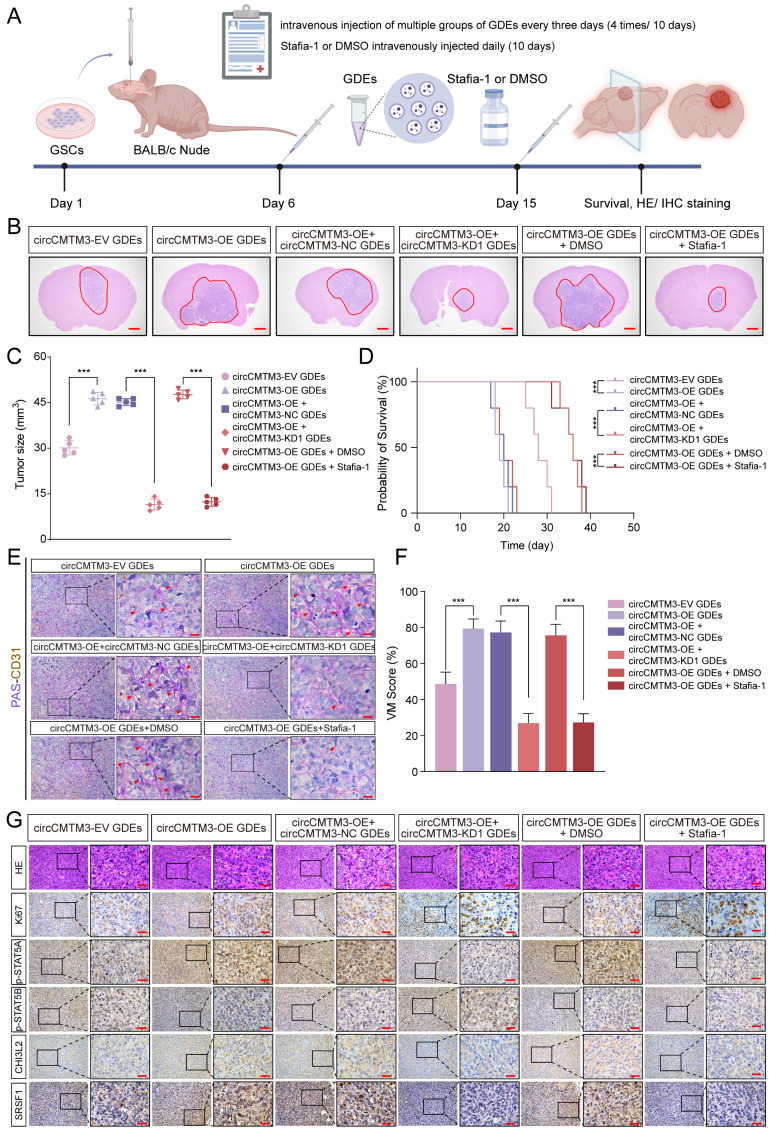
** Exosomal circCMTM3 promotes GBM malignant progression via facilitating VM formation in vivo. A** Schematic illustration of treatments for nude mice intracranially implanted with patient-derived GSCs. **B, C** Representative H&E staining (B) and correspond quantification (C) displaying tumor size in brain slices from different experimental groups (n = 5), Scale bars=1mm. **D** Kaplan-Meier analysis of mice from the indicated groups (n = 5). **E** Double staining showing the VM formation assessed by PAS and anti-CD31 immunohistochemical staining in tumor tissue. Red arrows indicating the VM tubular structures with PAS^+^/CD31^-^. Scale bars = 20 μm. **F** Assessment of VM scores in each group. **G** Representative H&E and immunohistochemical images demonstrating the morphological characteristics of GBM and expression of Ki-67, p-STAT5A, p-STAT5B, CHI3L2 and SRSF1 of tumor tissues from mice in different groups. Scale bar =50μm. Data are presented as means ± SD (three independent experiments). *p < 0.05; **p < 0.01; ***p < 0.001; ns, no significance.

To further investigate how exosomal circCMTM3 influences the rapid progression and VM formation of GBM in vivo, we comprehensively evaluated the pro-carcinogenic effects of GDEs packaged with abundant circCMTM3 in a mouse brain tumor xenografts model. Patient-derived GBM xenografts were established by stereotactic injection of GSCs into the striatum region of nude mice. Starting from day 6 after cell transplantation, tumor-bearing mice received multiple groups of GDEs via intravenous injection every three days. Additionally, we further validated the anti-VM effect of Stafia-1 targeting p-STAT5A (Figure 9A). Consistent with the in vitro findings, exosomal circCMTM3 treatment notably amplified the tumor burden in the orthotopic xenografts. In contrast, administration of Stafia-1 significantly restricted tumor volume and exhibited a positive anti-cancer effect (Figure 9B-C). Furthermore, Kaplan-Meier survival analysis indicated that exosomal circCMTM3 shortened overall survival, while treatment with Stafia-1 observably prolonged the survival time of tumor-bearing mice (Figure 9D). Subsequently, we performed pathology examination on mouse brain transplanted GSCs using PAS-CD31 double staining and observed that GDEs encapsulating overexpressed circCMTM3 led to the appearance of more VMs in the glioma tissues by in situ tumorigenesis assay, while Stafia-1 significantly reduced VM formation in tumor region (Figure 9E-F). Additionally, HE and IHC staining on in situ xenograft tumor specimens were conducted and the morphological characteristics of the circCMTM3/STAT5A/SRSF1 feedback loop were identified. Consistent with the conclusions from in vitro assays, the overexpression of exosomal circCMTM3 could trigger upregulation of p-STAT5A and the downregulation of p-STAT5B, leading to the amplification of CHI3L2 and SRSF1, which, in turn, promoted GBM progression characterized by the level of Ki-67 positivity. As expected, Stafia-1 reduced the expression of CHI3L2 and SRSF1 precisely (Figure 9G). In conclusion, we identified exosomal circCMTM3 as a factor promoting VM formation and accelerating the malignant progression of GBM in vivo experiments. We also discovered that Stafia-1 could exert anti-neovascularization activity by specifically blocking the operation of the circCMTM3/STAT5A/SRSF1/CHI3L2 feedback loop, highlighting the potential clinical application of Stafia-1.

To investigate the impact of exosomal circCMTM3-mediated vasculogenic mimicry (VM) on resistance to anti-angiogenic therapy in GBM, we conducted additional animal experiments simultaneously. After reviewing the literature, we found that the dynamin as a GTPase involved in endocytosis, whose inhibitor Dynasore can efficiently block the cellular uptake of exosomes both in vitro and in vivo^31^, ^32^. Therefore, in addition to exosome intervention in the intracranial tumor-bearing nude mouse model, we also evaluated the therapeutic effects of Bevacizumab and Dynasore. By measuring tumor volume (Figure S7A-B), recording the survival time of the tumor-bearing mice (Figure S7C), and assessing VM formation through PAS-CD31 staining (Figure S7D-E), we explored the contribution of exosomal circCMTM3-involved VM formation to resistance against anti-angiogenic therapy. Previous study has reported that autophagy induced by Bevacizumab treatment in GBM cells can promote VM formation, thereby leading to resistance to anti-angiogenic therapy^33^. Similarly, in our supplementary experiments, we found that Bevacizumab treatment failed to demonstrate an antitumor effect or delay GBM progression compared to the group administering with circCMTM3-overexpressed exosomes. In contrast, Dynasore, by inhibiting the endocytosis of exosomes by cells, exhibited significant anticancer activity by effectively blocking VM formation and slow tumor progression. These findings indicate that VM formation induced by exosomal circCMTM3 is a critical factor contributing to Bevacizumab resistance in GBM, and that blocking the cellular uptake of exosomes can effectively inhibit VM formation in GBM.

## Discussion

Tumor angiogenesis is an essential hallmark of cancer. In order to meet the continuous demands for proliferation, tumor cells undergo adaptive phenotypic switching under the influence of the local microenvironment to achieve rapid progression. In GBM, the formation of neovascular structures not only supplies oxygen and nutrients for tumor growth but also creates a vascular-rich microenvironment that often serves as a growth niche for GSCs^34^. Therefore, GSCs maintain their position at the top of the cellular hierarchy by regulating the production of vasculature components. However, anti-angiogenesis therapy has limited efficacy in treating nervous system tumors, and its underlying mechanisms remain unclear. Here, we discovered that DGCs undergo endothelial-like phenotype transformation regulated by exosomal circCMTM3 derived from GSCs, leading to VM formation, which provides strong support for the maintenance of GSCs both structurally and functionally.

Traditional anti-angiogenic therapies have primarily been recognized for their ability to inhibit endothelial cell proliferation and induce apoptosis, resulting in reduced vascular density and tumor tissue hypoxia^35^. However, these therapies have minimal impact on the formation of VM within the TME. This may also be a major reason why anti-angiogenic treatment for GBM fails to improve patient prognosis. Substantial evidence suggests that VM is an adverse prognostic factor in various malignant tumors^36^. Therefore, therapeutic strategies targeting VM, especially those with specific anti-VM targets, have been extensively explored in several solid malignancies. Previous studies have indicated the presence of VM structures within the parenchyma of GBM, where they specifically stimulate tumor proliferation. This study reveals the abundant presence of VM structures in GBM, consisting of DGCs, which enhance intercellular communication among various cell components within TME. Additionally, it offers potentially alternative therapeutic strategies for anti-vasculogenic treatment of GBM.

Extracellular vesicles, especially exosomes, play a pivotal role in facilitating intercellular communication between donor and recipient cells. Moreover, due to their highly stable structure and abundant content, circRNAs are frequently encapsulated within exosomes, enabling efficient intercellular transport. Additionally, exosomal circRNAs serve as valuable diagnostic and prognostic biomarkers in liquid biopsies^37^. Mounting evidence suggests that certain exosomal circRNAs exhibit aberrant expression in cancer cells and tumor tissues, thereby influencing tumor progression through mechanisms such as immune evasion, stimulation of angiogenesis, metabolic reprogramming, and drug resistance. For instance, exosome-derived circCCAR1 promotes CD8^+^ T-cell dysfunction and confers anti-PD1 resistance in hepatocellular carcinoma^38^. In GBM cases resistant to temozolomide (TMZ), the delivery of exosomal circWDR62 from resistant cells to sensitive cells imparts TMZ resistance while promoting a malignant tumor phenotype^39^. Furthermore, by regulating arachidonic acid metabolism, exosomal circRNA_101093 inhibits sensitivity to ferroptosis in lung adenocarcinoma^40^. These pro- or anti-cancer features of exosomal circRNAs pave the way for precise dynamic monitoring and targeted therapy of tumors. In this study, it was discovered that exosomal circCMTM3 exhibits cytokine-like or growth factor-like properties activating the JAK2/STAT5A signaling pathway non-classically, which promotes VM formation in GBM and accelerates malignant progression consequently. However, the specific mechanisms by which exosomal circRNAs derived from cellular components in the TME influence tumor cell fate remain incompletely understood, thus necessitating further investigation into elucidating the unique functions of these molecules in reshaping the TME.

The activation of the JAK-STAT signaling pathway is primarily mediated by two classical pathways: cytokine-receptor and growth factor-receptor tyrosine kinase (RTK) engagements. These pathways transmit activating signals that lead to the tyrosine phosphorylation and activation of JAKs associated with the receptors. Subsequently, downstream STATs are phosphorylated and activated, and the phosphorylated STATs translocate to the nucleus to regulate gene expression. Hence, the phosphorylation of STATs is crucial for their transcriptional function^41^. As an essential pathway for numerous fundamental cellular processes, the JAK/STAT pathway constitutes a rapid signal transduction module from the cell membrane to the nucleus and triggers the expression of various key mediators involved in cancer and inflammation. Increasing evidence suggests that dysregulation of the JAK/STAT pathway is associated with diverse cancers and autoimmune diseases^42^. STAT5, being a key molecule in the JAK/STAT pathway, has two homologous genes, STAT5A and STAT5B, which share 90% homology in their protein sequences. STAT5 is activated and exerts transcriptional activation function in response to various cytokine and growth factor signals. The phosphorylation modification of STAT5 following transcription often correlates with their active form and plays a positive regulatory role in the JAK/STAT pathway^43^. Studies have demonstrated that flavone isoxanthohumol inhibited VM formation by blocking IFN-γ, IL-4, and IL-6 dependent JAK/STAT signaling^44^. In this study, we identified a unique activation mode of STAT5A, which does not rely on cytokine and growth factor mediation but rather on the scaffolding effect provide by circCMTM3. Due to a 10% difference in the amino acid sequences between STAT5A and STAT5B, each interacts with a distinct fragment of circCMTM3. Consequently, there is rearrangement in spatial localization among STAT5A, STAT5B, and JAK2 leading to enhanced phosphorylation and activation of STAT5A, thereby amplifying neovascularization pathways in DGCs. Targeted blockade of p-STAT5A significantly reduces VM formation both in vivo and in vitro settings indicating anti-STAT5A as an encouraging therapeutic strategy against VM development in GBM.

Treatment-resistant diseases characterized by excessive activation of the JAK/STAT pathway, elevated serum levels of JAK-dependent cytokines, along with JAK/STAT mutations mainly include autoimmune diseases, malignant tumors, graft-versus-host disease (GVHD), and infectious diseases. Studies have reported that PIAS primarily inhibits STATs activity in the cell nucleus^45^. PIAS possesses small ubiquitin-related modifier (SUMO) E3 ligase activity, which can block the DNA-binding activity of STATs^46^. However, controversy remains regarding how E3 ligases regulate the activation level of the JAK/STAT axis through ubiquitination modification of STATs. Our findings reveal that CNOT4 ubiquitinates and promotes degradation of STAT5A/STAT5B in DGCs, while exosomal circCMTM3 from GSCs competitively binds to the RING domain of CNOT4 to block its E3 activity and maintain high expression of STAT5A/STAT5B, elucidating complex post-translational regulation of the JAK/STAT pathway in GBM progression. Additionally, the activation of the JAK/STAT pathway is accompanied by cross-talk between various cytokines and kinase signaling in the TME. Thus, a comprehensive study is necessary for understanding JAK2/STAT5A-mediated promotion of VM at tumor microecological level.

Chitinase-3-like protein 2 (CHI3L2), also known as YKL-39, is a secreted protein belonging to the chitinase-like protein (CLP) family^47^. Previous studies have shown that CHI3L2 is significantly upregulated in the relevant clinical pathological specimens of patients with osteoarthritis^48^, Alzheimer's disease^49^, multiple sclerosis^50^, and amyotrophic lateral sclerosis^51^. It is reported that CHI3L2 acts as potent monocyte chemoattractant and angiogenic stimulus promoting breast cancer metastasis during neoadjuvant chemotherapy and expectedly serves as novel target for anti-angiogenic therapy in breast cancer^52^. In GBM, CHI3L2 has been identified as a novel prognostic biomarker associated with immune infiltration markers in the TME^53^. In this study, we have provided the first evidence linking CHI3L2 with VM formation in GBM. This finding highlights the crucial role played by circCMTM3/STAT5A-mediated CHI3L2 overexpression in creating the necessary molecular conditions for VM development within the TME of GBM.

The serine/arginine-rich (SR) protein family is evolutionarily conserved and participates in both constitutive and alternative splicing processes of pre-mRNAs^54^. Several members of the SR family exhibit oncogenic properties in malignancy, with serine/arginine-rich splicing factor 1 (SRSF1) being particularly noteworthy due to its high expression in neoplastic tissues and ability to promote cellular phenotype transformation^55^. Previous studies have revealed that the expression of SRSF1 is regulated by complex mechanisms encompassing transcriptional, post-transcriptional, translational, and protein degradation processes^56^-^58^. Additionally, SRSF1 is typically involved in the formation of oncogenic signaling feedback loops, further amplifying its activity^59^. Our findings demonstrate that SRSF1 facilitates stable expression of exosomal circCMTM3 during cellular transport, indirectly leading to activation of STAT5A. Phosphorylated STAT5A translocated into the cell nucleus, where it binds to the SRSF1 promoter region and upregulates its expression levels, thereby constituting a positive feedback loop involving SRSF1/circCMTM3/STAT5A, which maintains the VM phenotype of DGCs.

## Conclusion

In summary, our study demonstrates that exosomal circCMTM3 derived from GSCs promotes the conversion and maintenance of DGCs into a VM phenotype, leading to the rapidly malignant progression of GBM. Mechanistically, circCMTM3 transported into DGCs acts as an antagonist to block the catalytic activity of the E3 ubiquitin ligase CNOT4, which prevents STAT5A/STAT5B from ubiquitinational degradation. Furtherly, circCMTM3 serves as a molecular scaffold, resulting in constitutive activation of STAT5A through a cytokine-independent, non-canonical mode. Furthermore, STAT5A transcriptionally upregulates the expression of CHI3L2 and SRSF1, which remodels the TME favorable for VM in GBM, and facilitates the accumulation and amplification of oncogenic signaling by triggering circCMTM3/STAT5A/SRSF1 positive feedback loop. Moreover, the upregulated exosomal circCMTM3, as a biomarker, indicates poor prognosis in glioma patients. Notably, exosomal circCMTM3 possessing growth factor-mimetic property non-classically activates the JAK2/STAT5A pathway promoting VM formation, which would be a potential vulnerability and orientation for GBM anti-vasculogenic therapies.

## Supplementary Material

Supplementary figures.

Supplementary tables.

## Abbreviations

NBT: Normal brain tissues
GSCs: Glioblastoma stem cells
GDEs: GSCs-derived exosomes
GBM: Glioblastoma
DGCs: differentiated glioma cells
circRNAs: Circular RNAs
TF: Transcription factor
RBPs: RNA binding proteins
FISH: fluorescence in situ hybridization
RT-qPCR: Real-time quantitative reverse transcription PCR
RIP: RNA immunoprecipitation
ChIP: Chromatin immunoprecipitation
IHC: Immunohistochemistry
MTS: 3-(4, 5-Dimethylthiazol-2-yl)-5-(3-carboxymethoxyphenyl)-2-(4-sulfophenyl)-2H tetrazolium
EdU: 5-Ethynyl-20-deoxyuridine
WHO: World Health Organization
STR: Short tandem repeat
GSEA: Gene set enrichment analysis
TCGA: The Cancer Genome Atlas
CGGA: Chinese Glioma Genome Atlas
