# Supplementary material for: Dual role of exosomal circCMTM3 derived from GSCs in impeding degradation and promoting phosphorylation of STAT5A to facilitate vasculogenic mimicry formation in glioblastoma
Source: Theranostics. 2024 Sep 3;14(14):5698–724. doi: 10.7150/thno.97057 (PMC11413784; doi:10.7150/thno.97057)
Supplement: Supplementary file 1 — Supplementary figures. [file thnov14p5698s1.pdf]

Supplementary figures

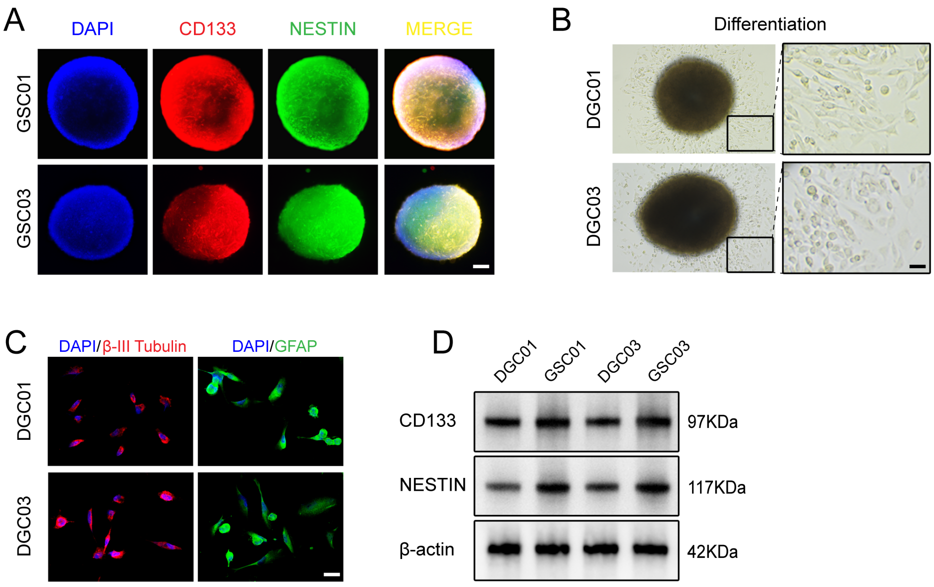

Figure S1 Molecular and morphological identification of GSC and DGCs. A Representative images of immunofluorescence staining showing the stemness markers expression of GSCs. Scale bars = 100 μm. **B** Representative images of the cellular morphology of DGCs. Scale bars = 50 μm. **C** Representative images of immunofluorescence staining showing the differentiational markers expression of DGCs. Scale bars = 50 μm. **D** Western blotting analysis of stemness markers on GSCs and DGCs.

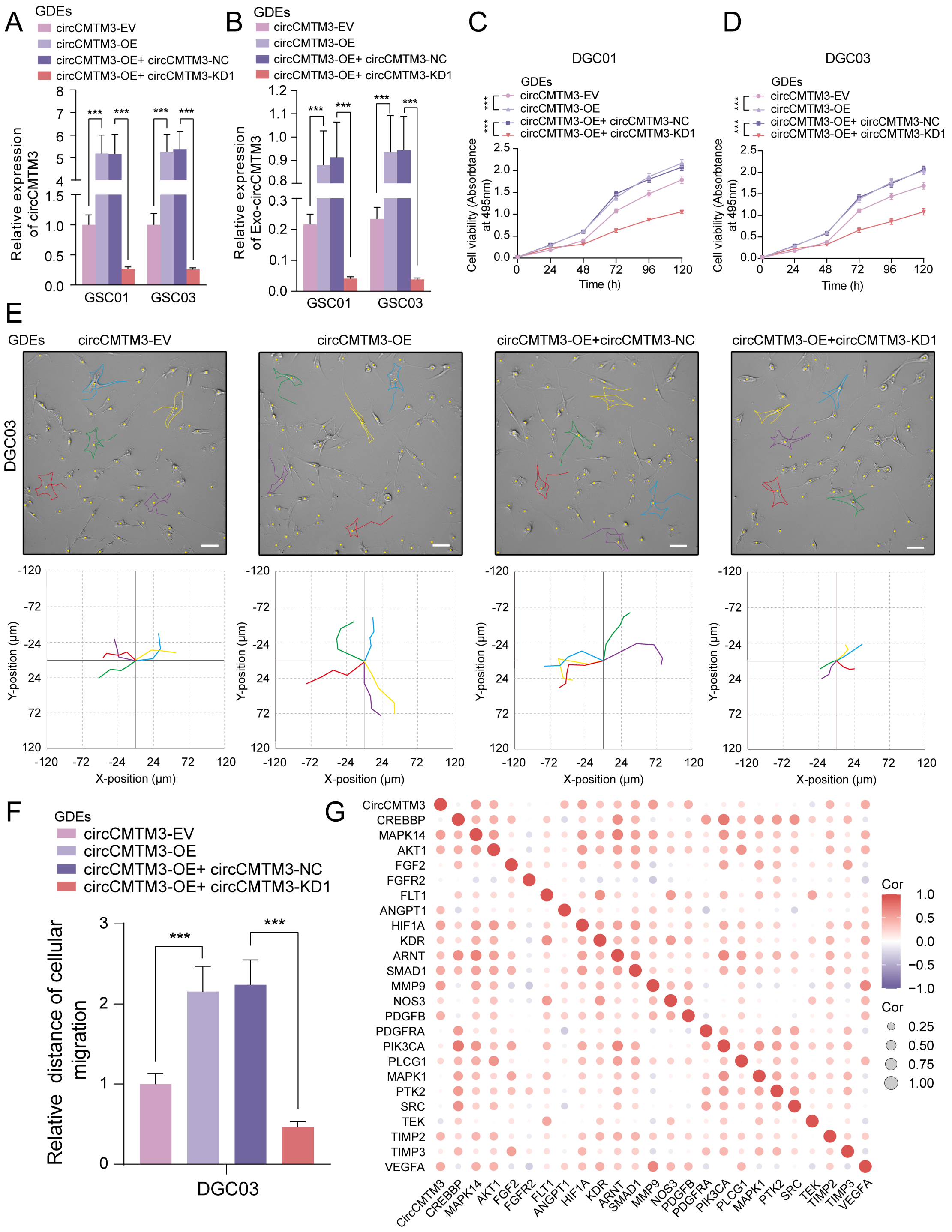

Figure S2 Exosomal circCMTM3 promotes proliferation and migration of DGCs and is corelated with angiogenesis pathway. A, B RT-qPCR assays displaying the expression of circCMTM3 in GSCs (A) and GDEs (B) after overexpression or knockdown. **C, D** MTS assays reveling the cell viabilities of DGC01 (C) and DGC03 (D) by incubating with different groups of GDEs. **E, F** Representative images (E) visualized in Hstudio and quantification (F) of the DGC03 migration ability (n = 5). Scale bars = 50 μm. **G** Correlation of exosomal circCMTM3 expression with angiogenesis pathway genes. Data are presented as means ± SD (three independent

experiments). *$p < 0.05$; **$p < 0.01$; ***$p < 0.001$; ns, no significance.

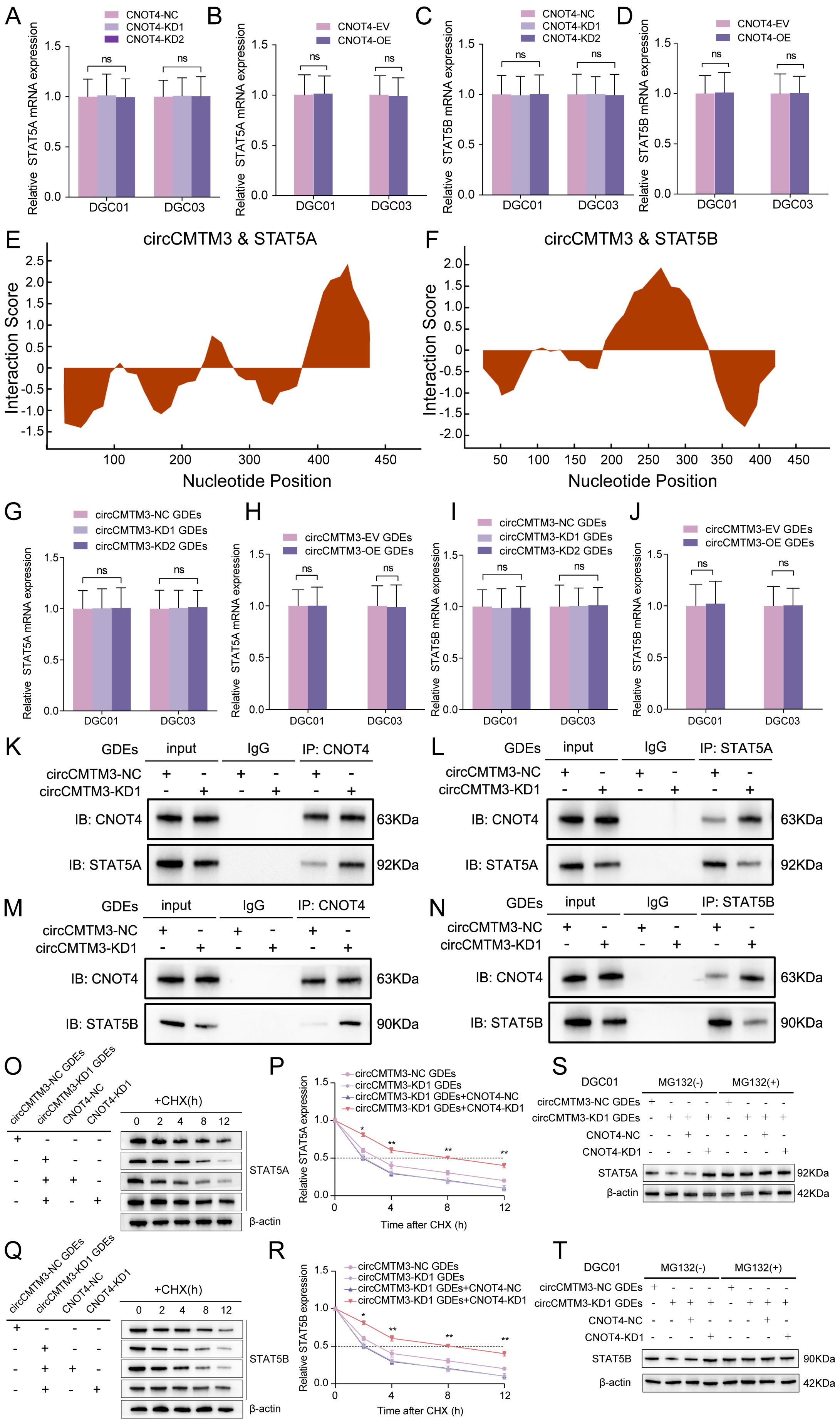
A
CNOT4-NC
CNOT4-KD1
CNOT4-KD2
Relative STAT5A mRNA expression
ns
DGC01
DGC03
B
CNOT4-EV
CNOT4-OE
Relative STAT5A mRNA expression
C
Relative STAT5B mRNA expression
D
E
circCMTM3 & STAT5A
Interaction Score
Nucleotide Position
F
circCMTM3 & STAT5B
G
circCMTM3-NC GDEs
circCMTM3-KD1 GDEs
circCMTM3-KD2 GDEs
H
circCMTM3-EV GDEs
circCMTM3-OE GDEs
I
J
K
GDEs
input
IgG
IP: CNOT4
circCMTM3-NC
circCMTM3-KD1
IB: CNOT4
IB: STAT5A
63KDa
92KDa
L
IP: STAT5A
M
IB: STAT5B
90KDa
N
IP: STAT5B
O
+CHX(h)
CNOT4-NC
CNOT4-KD1
STAT5A
β-actin
P
circCMTM3-KD1 GDEs+CNOT4-NC
circCMTM3-KD1 GDEs+CNOT4-KD1
Relative STAT5A expression
Time after CHX (h)
S
DGC01
MG132(-)
MG132(+)
42KDa
Q
STAT5B
R
Relative STAT5B expression
T

Figure S3 Exosomal circCMTM3 upregulates STAT5A/B expressional level via inhibiting the ubiquitin- proteasomal pathway. A-D RT-qPCR assays showing the mRNA expression of STAT5A and STAT5B in CNOT4 -overexpressed or silencing DGCs. **E, F** CircCMTM3 can bind to STAT5A and STAT5B proteins via CatRAPID prediction. **G-J** RT-qPCR assays displaying the mRNA expression of STAT5A and STAT5B in DGCs with treatment of different groups of GDEs. **K-N** Co-IP assays illustrating interaction efficiency of CNOT4 and STAT5A (K, L) as well as CNOT4 and STAT5B (M, N) in DGCs under the condition of exosomal circCMTM3 knockdown. **O-R** Western blotting analysis showing STAT5A and STAT5B expression in CNOT4-silenced DGCs with treatment by GDEs containing downregulated circCMTM3 and CHX (50 μg/ml) (O, Q), meanwhile, quantitative analysis on STAT5A and STAT5B expression half-life time ($t_{1/2}$) reflecting degradation rates (P, R). **S, T** Western blotting assays showing STAT5A (S) and STAT5B (T) expression in CNOT4-silenced DGC01 treated with or without MG-132(50 μM) after incubating with circCMTM3-downregulated GDEs. Data are presented as means ± SD (three independent experiments). $*p < 0.05$; $**p < 0.01$; $***p < 0.001$; ns, no significance.

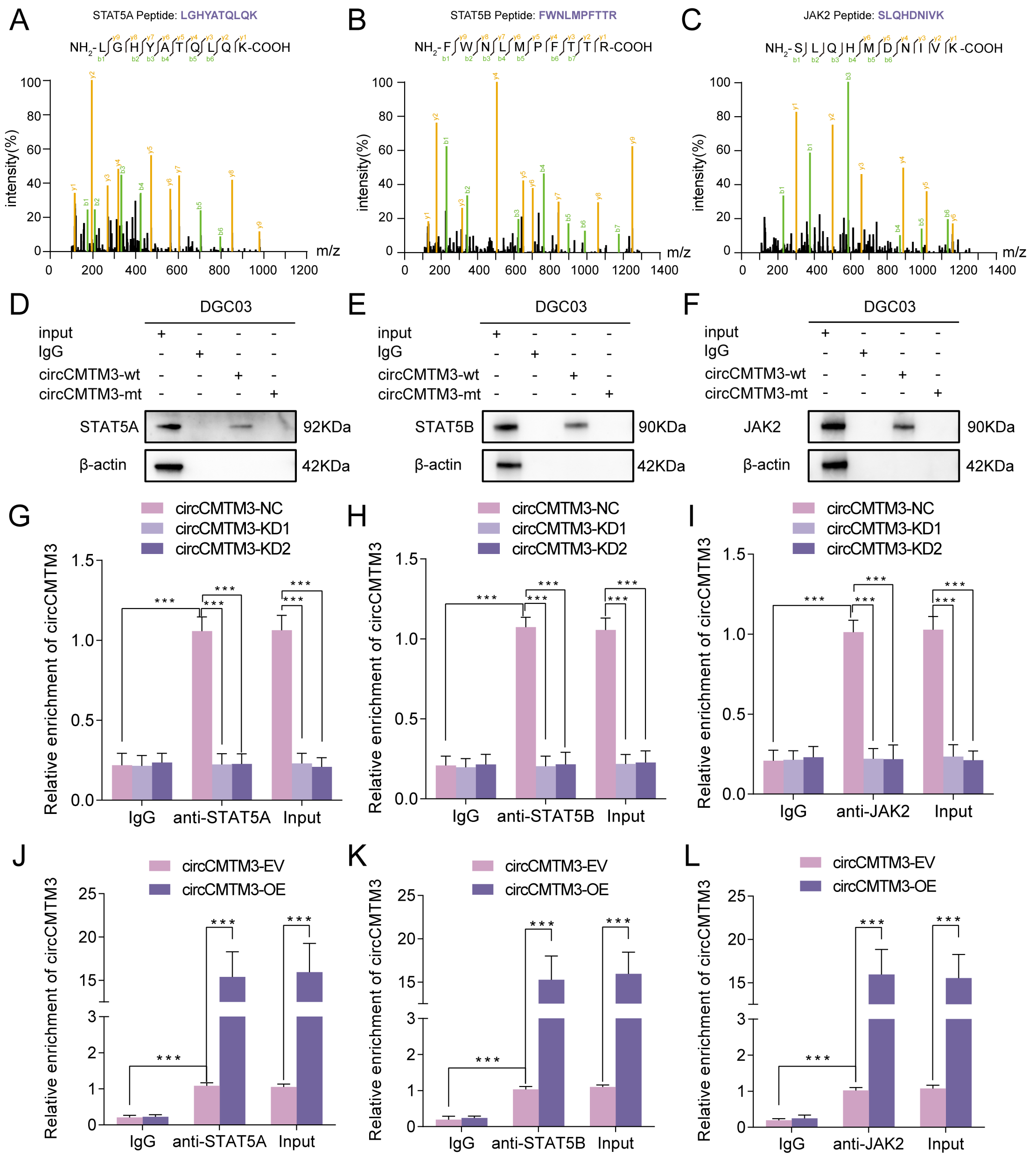

Figure S4 Exosomal circCMTM3 interacts with STAT5A, STAT5B and JAK2 respectively in DGCs. A-C LC-MS/MS spectrum showing the STAT5A, STAT5B and JAK2 peptides pulled down by circCMTM3 probe respectively. **D-F** RNA pull-down assay to validate the interaction between exosomal circCMTM3 and JAK2, STAT5A, and STAT5B respectively in DGC03. **G-L** RIP assays showing anti-STAT5A (G, J), anti-STAT5B (H, K) and anti-JAK2 (I, L) treatment caused exosomal circCMTM3 enrichment in DGC03 by treatment with different groups of GDEs. Data are presented as means ± SD (three independent experiments). *p < 0.05; **p < 0.01; ***p < 0.001; ns, no significance.

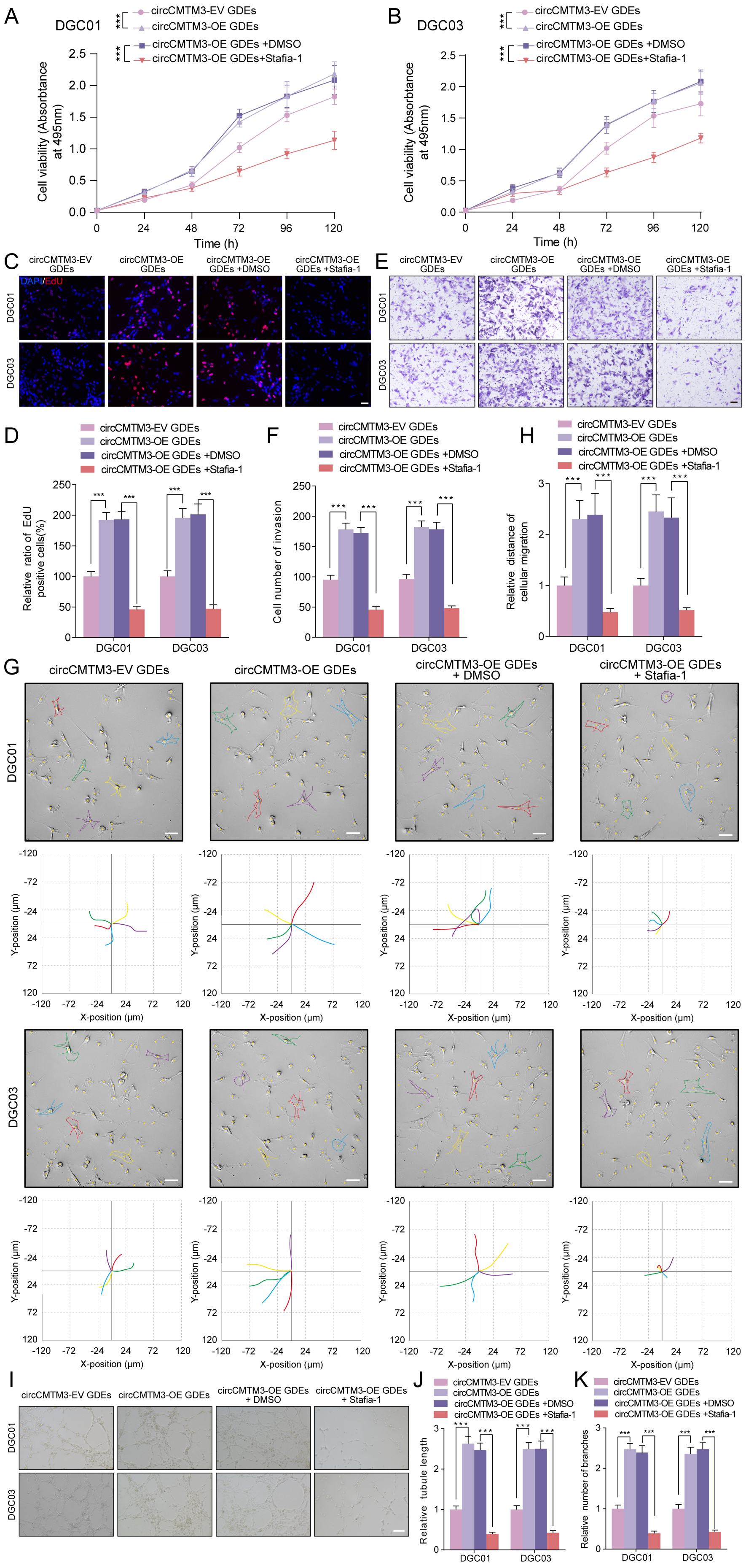

A
DGC01
circCMTM3-EV GDEs
circCMTM3-OE GDEs
circCMTM3-OE GDEs +DMSO
circCMTM3-OE GDEs+Stafia-1
***
***
Cell viability (Absorbance at 495nm)
Time (h)
B
DGC03
C
DAPI/EdU
circCMTM3-EV GDEs
circCMTM3-OE GDEs
circCMTM3-OE GDEs +DMSO
circCMTM3-OE GDEs +Stafia-1
DGC01
DGC03
E
D
Relative ratio of EdU positive cells(%)
F
Cell number of invasion
H
Relative distance of cellular migration
G
circCMTM3-OE GDEs + DMSO
circCMTM3-OE GDEs + Stafia-1
Y-position (μm)
X-position (μm)
I
J
Relative tubule length
K
Relative number of branches

Figure S5 Exosomal circCMTM3 induces VM formation in vitro via upregulating p-STAT5A expression. A, B MTS assays assessing the cell viabilities of DGC01 (A) and DGC03 (B) incubated with circCMTM3-EV GDEs and circCMTM3-OE GDEs with DMSO or Stafia-1. **C, D** EdU assays (C) and quantified analysis (D) showing the proliferation of DGC01 and DGC03 with the indicated treatments. Scale bars = 100 μm. **E, F** Representative photographs (E) and quantification (F) of the Transwell assay of DGCs from each group. Scale bars = 50 μm. **G, H** Representative photographs (G) and quantification of relative migration distance (H) exhibiting the migration ability of DGC01 and DGC03 detected by HoloMonitor and visualized in Hstudio (n = 5) in each group. Scale bars = 50 μm. **I-K** Representative images (I) and quantification (J, K) showing tube formation of DGCs. Scale bars=100μm. Data are presented as means ± SD (three independent experiments). *$p < 0.05$; **$p < 0.01$; ***$p < 0.001$; ns, no significance.

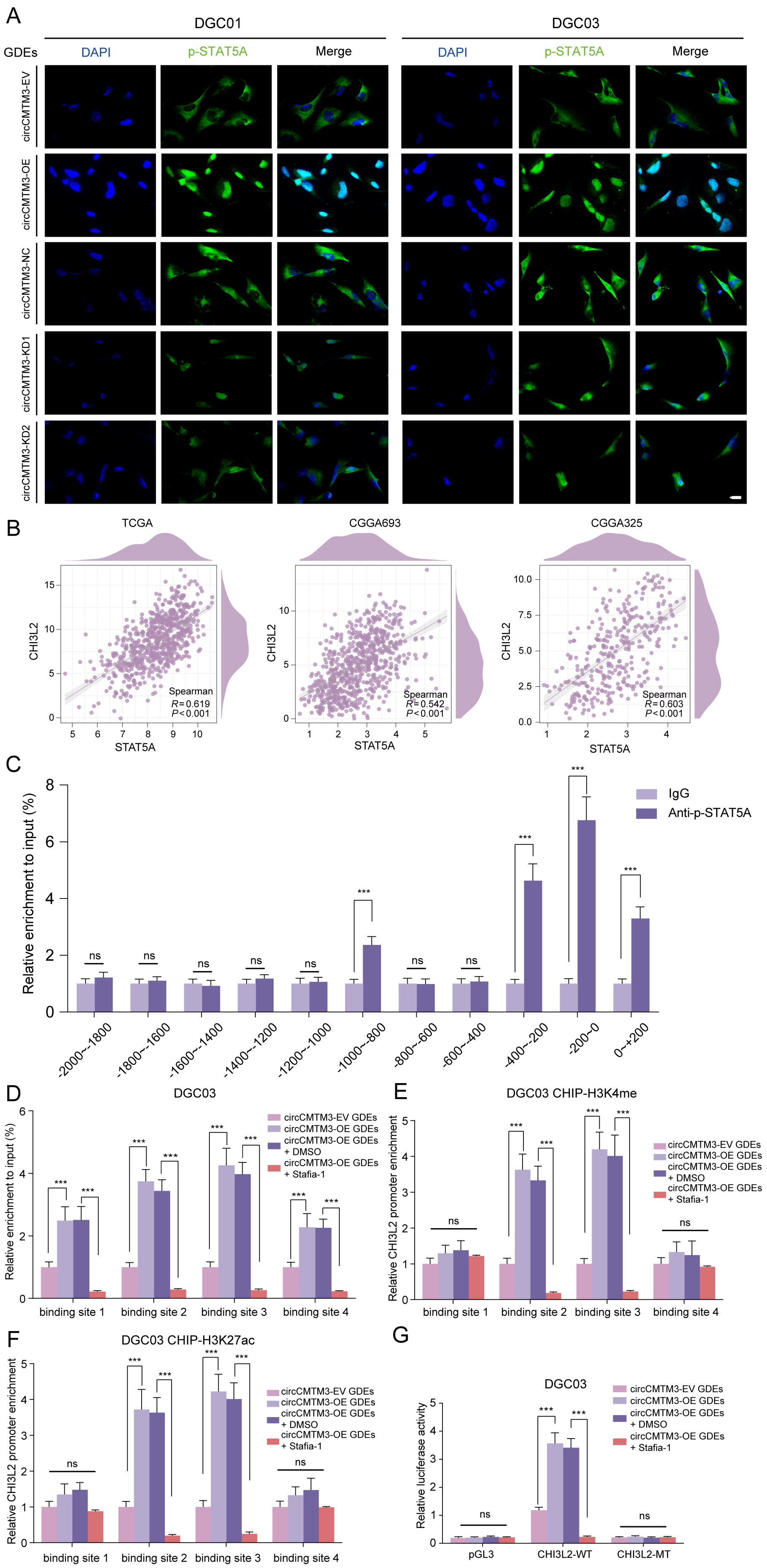
A
DGC01
DGC03
GDEs
DAPI
p-STAT5A
Merge
circCMTM3-EV
circCMTM3-OE
circCMTM3-NC
circCMTM3-KD1
circCMTM3-KD2
B
TCGA
CGGA693
CGGA325
CHI3L2
STAT5A
Spearman
R=0.619
P<0.001
R=0.542
R=0.603
C
Relative enrichment to input (%)
IgG
Anti-p-STAT5A
-2000~-1800
-1800~-1600
-1600~-1400
-1400~-1200
-1200~-1000
-1000~-800
-800~-600
-600~-400
-400~-200
-200~0
0~+200
D
DGC03
Relative enrichment to input (%)
circCMTM3-EV GDEs
circCMTM3-OE GDEs
circCMTM3-OE GDEs + DMSO
circCMTM3-OE GDEs + Stafia-1
binding site 1
binding site 2
binding site 3
binding site 4
E
DGC03 CHIP-H3K4me
Relative CHI3L2 promoter enrichment
F
DGC03 CHIP-H3K27ac
G
DGC03
Relative luciferase activity
pGL3
CHI3L2-WT
CHI3L2-MT

Figure S6 Exosomal circCMTM3 activates expression of CHI3L2 in DGCs. A Representative images of immunofluorescence staining showing the expression and subcellular distribution in DGCs with knockdown or overexpression exosomal circCMTM3 treatment. Scale bars = 50 μm. **B** Correlation analysis of STAT5A and CHI3L2 based on TCGA-glioma, CGGA325 and CGGA693 datasets. **C** ChIP analyses of the STAT5A-bound promoter regions of CHI3L2. The corresponding isotype IgG was used as a negative control. **D** Quantification of ChIP assay showing the STAT5A binding sites in the CHI3L2 promoters in DGC03 treated with circCMTM3-OE GDEs with or without Stafia-1intervention. **E, F** ChIP-qPCR detecting H3K4me (E) and H3K27ac (F) enrichment in the CHI3L2 promoters in DGC03 with indicated intervention. **G** The Dual-luciferase reporter assays revealing the luciferase promoter activities of CHI3L2 with treatment by different group GDEs and Stafia-1 or not in DGC03. Data are presented as means ± SD (three independent experiments). *p < 0.05; **p < 0.01; ***p < 0.001; ns, no significance.

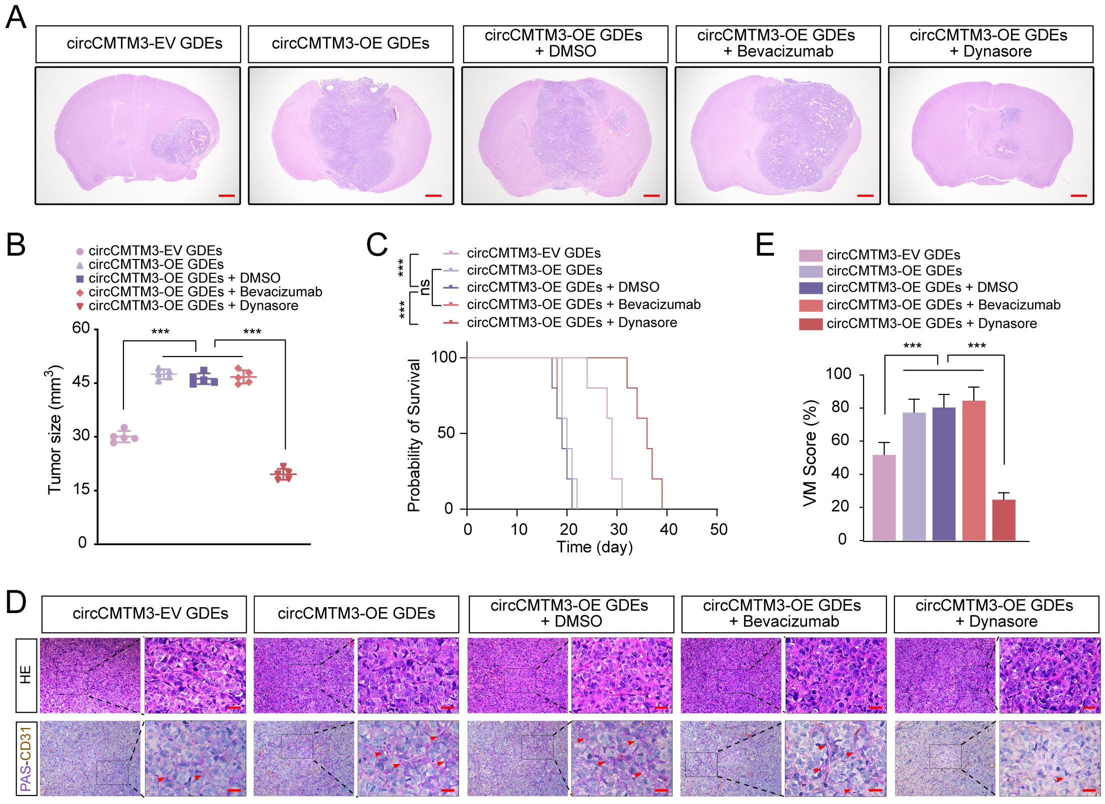

Figure S7 Exosomal circCMTM3 mediates resistance to anti-angiogenic therapy by promoting VM formation in GBM. A, B Representative H&E staining (A) and correspond quantification (B) displaying tumor size in brain slices from different experimental groups (n = 5), Scale bars = 1 mm. **C** Kaplan–Meier analysis of mice from the indicated groups (n = 5). **D** Representative H&E staining images demonstrating the morphological characteristics of GBM. Double staining showing the VM formation assessed by PAS and anti-CD31 immunohistochemical staining in tumor tissue. Red arrows indicating the VM tubular structures with $PAS^{+}/CD31^{-}$. Scale bars = 20 μm. **E** Assessment of VM scores in each group. Data are presented as means ± SD (three

independent experiments). *p < 0.05; **p < 0.01; ***p < 0.001; ns, no significance.
